# Supplementary material for: Study protocol for the antidepressant advisor (ADeSS): a decision support system for antidepressant treatment for depression in UK primary care: a feasibility study
Source: BMJ Open. 2020 May 24;10(5):e035905. doi: 10.1136/bmjopen-2019-035905 (PMC7252992; doi:10.1136/bmjopen-2019-035905)
Supplement: Supplementary data [file bmjopen-2019-035905supp001.pdf]

SUPPLEMENTARY FILE 1

Study Protocol for The Antidepressant Advisor (ADeSS): A Decision Support System for Antidepressant Treatment for Depression in UK primary care - a feasibility study

Short Title: The Antidepressant Advisor

Supplementary Methods

EMIS Eligibility Tool Criteria

1) current or recent prescription (in the last 2 months) of any of the following antidepressants listed: citalopram, fluoxetine, sertraline, escitalopram, paroxetine, venlafaxine, or duloxetine
2) previous prescription of at least one other antidepressant out of the same list of antidepressants
3) exclude patients for whom both escitalopram and sertraline have already been prescribed
4) no previous prescription of mirtazapine or vortioxetine
5) no mania/hypomania/bipolar (or synonyms), psychosis/schizophrenia (or synonyms), or drug abuse/misuse/dependence (or synonyms), or alcohol abuse/misuse/dependence or synonyms
6) age≥18 years
7) not pregnant
8) not given birth in last 6 months.

Decision Support Tool Rationale & Details

The decision chart for the decision support tool is presented in Figure 1 below. A network meta-analysis, not including vortioxetine, showed that sertraline and escitalopram are superior in terms of acceptability and efficacy compared to other antidepressants [1]. Since sertraline is among the first–line options recommended by NICE, if patients tolerate serotonin reuptake inhibitors (SSRI), the algorithm starts with recommending escitalopram for those patients where two NICE recommended first-line treatments have failed including sertraline, but starts with sertraline should it not have been prescribed before. For patients who have not tolerated SSRIs (verified by GP), the algorithm makes alternative suggestions. Mirtazapine is among the most efficacious antidepressants [1] and has a different side effect profile to SSRIs which is why it is suggested for these patients. Because of its 75% risk in inducing weight gain and thereby increasing the risk of metabolic syndrome[2], the algorithm suggests

Harrison P, *et al. BMJ Open* 2020; 10:e035905. doi: 10.1136/bmjopen-2019-035905

vortioxetine in those patients who are at an increased risk of metabolic syndrome (Body Mass Index(BMI)>25, HbA1c increased), because of its low weight-gain-induction risk[3]. Vortioxetine has SSRI properties, but is less likely to induce typical side effects, apart from nausea [3]. A recent NICE health technology appraisal [4]has given a positive recommendation for using vortioxetine in patients whose depressive episode has responded inadequately to two antidepressants. The REVIVE trial [3] showed the benefits of vortioxetine in patients with current MDD who had not responded to SSRI or dual-reuptake inhibitor monotherapy. The appraisal also concluded that vortioxetine may have a better overall safety profile than other antidepressants. For those patients tolerating SSRIs who do not respond to escitalopram and are at an increased metabolic syndrome risk, the algorithm therefore suggests vortioxetine.

Venlafaxine, which is among the most efficacious medications[1], was omitted from the algorithm, because of its higher risk of death in overdose which limits its use in primary care according to the Medicines and Healthcare Regulatory Agency [5]guidance and most GPs we discussed this with are not prepared to prescribe it at the higher doses at which it acts as a noradrenaline reuptake inhibitor. Duloxetine was excluded as an alternative as it was less efficacious in a network meta-analysis [1].

For escitalopram non-responders who are at low metabolic syndrome risk, the algorithm suggests mirtazapine as a next step. For mirtazapine non-responders, a switch to vortioxetine is suggested as a next step. For vortioxetine non-responders during the second or third step, a psychiatric referral to the study psychiatrist is suggested, who could then recommend higher side effect burdened alternatives such as high-dose venlafaxine or augmentation strategies according to NICE guidelines. Agomelatine has been omitted from the algorithm despite its suitability for patients who are not tolerating SSRIs, because there is no positive HTA recommendation for it and because it requires liver enzyme checks [6].

Data Management

Data will be entered electronically into a password-protected database and stored separately from identifiable information. Data validation will be applied to ensure that entered data follows the correct format and is within a valid range. Data will be stored electronically in the master folder and will only be accessed by members of the research team. Data will be

Harrison P, *et al. BMJ Open* 2020; 10:e035905. doi: 10.1136/bmjopen-2019-035905

backed up by the research associate every time the database is added to on an external hard drive.

Harms

An adverse event (AE) is any untoward medical occurrence in a patient or healthy volunteer, which does not necessarily have a causal relationship with the study procedures. A Serious Adverse Event (SAE) is any undesirable medical occurrence in a participant that is life threatening, requires hospitalisation, results in persistent/significant disability/incapacity, causes a congenital anomaly/ birth defect or results in death.

These events may be spontaneously revealed by the subject or observed by the GP and will be recorded regardless of whether the GP believes them to be related to the intervention. The following aspects of AEs/SAEs will be assessed: symptom, possible diagnosis or syndrome, date and duration of AE/SAE, causal relationship with investigation (definite / probable / possible / unlikely / not related), outcome (resolved / resolving / not resolved / resolved with sequelae / fatal / unknown), treatment of event, if applicable (e.g. any medication as well as any other medical measures), actions taken regarding assessment.

GPs will be asked to report SAEs within 24 hours to Lambeth CCG and the PI via email or phone without details which are then followed up with a more detailed report. SAEs which according to the GP are clearly unrelated to the study intervention (e.g. hospitalization for an unrelated illness) and AEs expected when treating depression[i], even if there is a possible relation to the intervention, are exempt from this immediate reporting and will instead be reported together with other AEs at the earliest opportunity (at least quarterly). An executive committee formed of two members of the TSC (one independent) will discuss any AEs/SAEs prior to each TSC then report any concerns back to the TSC. Blinded members of the TSC will be asked to leave the room for the discussion of AEs/SAEs in order to remain blinded. Medically important events or SAEs will be assessed by a medically qualified doctor in the study team and discussed with the TSC. If possible, blinded doctors will assess serious or medically important events, whereas other AEs may be classified by a non-blinded doctor. Classification into SUSAR vs. expected ones will be based on the most updated versions of the summary product characteristics of the prescribed drugs on https://www.medicines.org.uk/emc/. SUSARs would be reported to the sponsor and ethics

committee. GPs will be asked to document all other AEs and reasons for withdrawing from the study on EMIS.

Consent

Consent and screening will be carried out in two stages. First, patients identified by the EMIS eligibility tool will be asked to provide written consent to participate in the online pre-screening assessment, with phone and postal options also offered. Second, patients found to be eligible at pre-screening will be invited to the baseline assessment where they will be asked to provide written consent to participate in the feasibility trial. Each participant will receive an oral and written explanation of the purposes, procedures, and risks of this study. Written consent will be obtained after explaining the goals, procedures, risks (and discomforts), benefits, and nature of the study to patients. Only adult volunteers and patients who are capable of understanding the study procedures will be allowed to sign the consent forms.

Participants can withdraw from the trial at any time and this will be made clear in the information sheets and consent forms. The reason for withdrawal does not need to be provided.

Dissemination Policy

Reporting bias will be avoided by a priori registration in a public trial register in addition to publication of the current trial protocol as the research is ongoing. All members of the TSC will have the opportunity to review and have input into the current protocol. The results of the study will be published in a pre-publication archive within one year after completion.

Harrison P, *et al. BMJ Open* 2020; 10:e035905. doi: 10.1136/bmjopen-2019-035905

References

1. Cipriani A, Furukawa TA, Salanti G et al. Comparative efficacy and acceptability of 12 new-generation antidepressants: a multiple-treatments meta-analysis. Lancet 2009;373(9665):746-58.
2. Masand PS, Gupta S. Long-term side effects of newer-generation antidepressants: SSRIS, venlafaxine, nefazodone, bupropion, and mirtazapine. Ann Clin Psychiatry 2002;14(3):175-82.
3. Montgomery SA, Nielsen RZ, Poulsen LH, Häggström L. A randomised, double-blind study in adults with major depressive disorder with an inadequate response to a single course of selective serotonin reuptake inhibitor or serotonin–noradrenaline reuptake inhibitor treatment switched to vortioxetine or agomelatine. Human Psychopharmacology: Clinical and Experimental 2014;29(5):470-482.
4. NICE. Vortioxetine for treating major depressive episodes - Technology appraisal guidance. 2015.
5. (MHRA). MaHRA. Selective serotonin reuptake inhibitors (SSRIs) and serotonin and noradrenaline reuptake inhibitors (SNRIs): use and safety. . 2014.
6. Agomelatine (Valdoxan): risk of liver toxicity. . Medicines and Healthcare Regulatory Agency (MHRA). 2014.

[i] These include non-fatal suicide attempt, non-suicidal self-harm-related injury, non-fatal traffic accident under medication influence, treatment-emergent [hypo-]mania with associated non-fatal complications, non-fatal somatic or anxiety-related complaints leading to A&E visits).

Harrison P, *et al. BMJ Open* 2020; 10:e035905. doi: 10.1136/bmjopen-2019-035905
