# Supplementary material for: Study protocol for the antidepressant advisor (ADeSS): a decision support system for antidepressant treatment for depression in UK primary care: a feasibility study
Source: BMJ Open. 2020 May 24;10(5):e035905. doi: 10.1136/bmjopen-2019-035905 (PMC7252992; doi:10.1136/bmjopen-2019-035905)
Supplement: Supplementary data [file bmjopen-2019-035905supp002.pdf]

SUPPLEMENTARY FILE 2

Participant Identification Number for this trial: **ADeSS-S1-**

Date of Birth:

CONSENT FORM

Study Title: Antidepressant Advisor – Study 1
Name of Chief Investigator: Dr Roland Zahn

Please initial:

1. I confirm that I have read and understand the information sheet dated 24 Jan 2018 (version 2) for the above study. I have had the opportunity to consider the information, ask questions and have had these answered satisfactorily.	☐
2. I understand that my participation is voluntary and that I am free to withdraw at any time without giving any reason, without my medical care or legal rights being affected.	☐
3. I understand that relevant sections of my medical notes and data collected during the study may be looked at by individuals from the research team. I give permission for these individuals to have access to my records.	☐
4. I agree to my GP/responsible clinician being informed of my participation in the study and that they receive a report on the diagnostic assessment carried out.	☐
5. I agree to be contacted again in the future by members of the research team of this study and to be asked whether I would be willing to participate in another study.	☐
6. I agree that a video/audio recording of the testing session may be made for training purposes of members of the research team supervised by external experts, who are required to keep the contents confidential (This is optional).	
7. I agree to take part in the above study.	☐

Name of Participant	Date	Signature
Name of Person taking consent	Date	Signature

One form for participant, one form for researcher site file; the original to be kept in medical notes.

Harrison P, *et al. BMJ Open* 2020; 10:e035905. doi: 10.1136/bmjopen-2019-035905
