# Supplementary material for: Study protocol for the antidepressant advisor (ADeSS): a decision support system for antidepressant treatment for depression in UK primary care: a feasibility study
Source: BMJ Open. 2020 May 24;10(5):e035905. doi: 10.1136/bmjopen-2019-035905 (PMC7252992; doi:10.1136/bmjopen-2019-035905)
Supplement: Author's manuscript [file bmjopen-2019-035905.draft_revisions.pdf]

BMJ Open is committed to open peer review. As part of this commitment we make the peer review history of every article we publish publicly available.

When an article is published we post the peer reviewers' comments and the authors' responses online. We also post the versions of the paper that were used during peer review. These are the versions that the peer review comments apply to.

The versions of the paper that follow are the versions that were submitted during the peer review process. They are not the versions of record or the final published versions. They should not be cited or distributed as the published version of this manuscript.

BMJ Open is an open access journal and the full, final, typeset and author-corrected version of record of the manuscript is available on our site with no access controls, subscription charges or pay-per-view fees (http://bmjopen.bmj.com).

If you have any questions on BMJ Open's open peer review process please email info.bmjopen@bmj.com

BMJ Open

Study Protocol for The Antidepressant Advisor (ADeSS): A Decision Support System for Antidepressant Treatment for Depression in UK primary care - a feasibility study

Journal:	*BMJ Open*
Manuscript ID	bmjopen-2019-035905
Article Type:	Protocol
Date Submitted by the Author:	22-Nov-2019
Complete List of Authors:	Harrison, Phillippa; Centre for Affective Disorders, Department of Psychological Medicine, Institute of Psychiatry, Psychology & Neuroscience, King's College London<br>Carr, Ewan ; Department of Biostatistics and Health Informatics, Institute of Psychiatry, Psychology & Neuroscience, King's College London<br>Goldsmith, Kimberley; Department of Biostatistics and Health Informatics, Institute of Psychiatry, Psychology & Neuroscience, King's College London<br>Young, Allan; Centre for Affective Disorders, Department of Psychological Medicine, Institute of Psychiatry, Psychology & Neuroscience, King's College London<br>Ashworth, Mark; Department of Population Health Sciences, King's College London<br>Fennema, Diede; Centre for Affective Disorders, Department of Psychological Medicine, Institute of Psychiatry, Psychology & Neuroscience, King's College London<br>Barrett, Barbara; Department of Health Services & Population Research, Institute of Psychiatry, Psychology & Neuroscience, King's College London<br>Zahn, Roland; Centre for Affective Disorders, Department of Psychological Medicine, Institute of Psychiatry, Psychology & Neuroscience, King's College London
Keywords:	Depression & mood disorders < PSYCHIATRY, PRIMARY CARE, Adult psychiatry < PSYCHIATRY

SCHOLARONE™
Manuscripts

I, the Submitting Author has the right to grant and does grant on behalf of all authors of the Work (as defined in the below author licence), an exclusive licence and/or a non-exclusive licence for contributions from authors who are: i) UK Crown employees; ii) where BMJ has agreed a CC-BY licence shall apply, and/or iii) in accordance with the terms applicable for US Federal Government officers or employees acting as part of their official duties; on a worldwide, perpetual, irrevocable, royalty-free basis to BMJ Publishing Group Ltd ("BMJ") its licensees and where the relevant Journal is co-owned by BMJ to the co-owners of the Journal, to publish the Work in this journal and any other BMJ products and to exploit all rights, as set out in our licence.

The Submitting Author accepts and understands that any supply made under these terms is made by BMJ to the Submitting Author unless you are acting as an employee on behalf of your employer or a postgraduate student of an affiliated institution which is paying any applicable article publishing charge ("APC") for Open Access articles. Where the Submitting Author wishes to make the Work available on an Open Access basis (and intends to pay the relevant APC), the terms of reuse of such Open Access shall be governed by a Creative Commons licence – details of these licences and which Creative Commons licence will apply to this Work are set out in our licence referred to above.

Other than as permitted in any relevant BMJ Author's Self Archiving Policies, I confirm this Work has not been accepted for publication elsewhere, is not being considered for publication elsewhere and does not duplicate material already published. I confirm all authors consent to publication of this Work and authorise the granting of this licence.

For peer review only

Abstract

Introduction: The Antidepressant Advisor Study is a feasibility trial of a computerised decision-support tool which uses an algorithm to provide antidepressant treatment guidance for General Practitioners (GPs) in the UK primary care service. The tool is the first in the UK to implement national guidelines on antidepressant treatment guidance into a computerised decision support tool.

Methods and analysis: The study is a parallel group, cluster-randomised controlled feasibility trial where participants are blind to treatment allocation. GPs were assigned to two treatment arms: 1) treatment as usual, 2) computerised decision support tool to assist with antidepressant choices. The study will assess recruitment and lost to follow-up rates, GP satisfaction with the tool, and impact on health service use. A meaningful long-term roll-out unit cost will be calculated for the tool and service use data will be collected at baseline and follow-up to inform a full economic evaluation of a future trial.

Ethics and dissemination: The study has received NHS ethical approval from the London - Camberwell St Giles Research Ethics Committee (ref:17/LO/2074). The trial was pre-registered in the Clinical Trials.gov registry. The results of the study will be published in a pre-publication archive within one year of completion of the last follow-up assessment.

Trial registration number: ClinicalTrials.gov Identifier: NCT03628027

Strengths and limitations of this study:

- A strength of the study is integration of a decision support tool into an existing UK primary care computer system to reduce the burden on GPs of adopting a new treatment system
- A further strength is that the study allows patients to be treated by their regular GP practice which reduces the burden on patients
- A limitation of the study is the potential for selection bias towards those with less severe depression who are well enough to attend study assessments

For peer review only - http://bmjopen.bmj.com/site/about/guidelines.xhtml

Introduction

Background & Rationale

Depression is a leading cause of disability world-wide [1] with approximately two million adults in the UK experiencing an episode each week [2]. Population-based studies show that only 50% of people with major depressive disorder (MDD) are treated [3; 4] and more than half of these receive inadequate treatment [4]. Furthermore, less than one third of patients fully recover after treatment with a standard antidepressant, such as citalopram [5], and only 40% of patients recover after accessing the National Health Service (NHS) Improving Access to Psychological Therapies (IAPT) programme [6]. The UK National Institute for Health and Care Excellence (NICE) recommends three first-line antidepressants with similar mechanism of action (fluoxetine, sertraline, and citalopram) [7]. Second- and third-line treatments are recommended by NICE, but, due to a lack of sequenced guidance, it is unclear whether and when GPs should prescribe them [7]. Overall, unlike for the management of other long-term conditions, there is no structured disease management for antidepressant prescribing in UK primary care [8; 9] and patients' access to second- and third-line antidepressants is variable. National prescription data show frequent and rising use of certain second-line antidepressants such as venlafaxine and mirtazapine without clear decision strategies [10]. A lack of structure in treatment decision-making and poor implementation of existing guidelines could exacerbate the suffering caused by insufficiently treated depression [4]. Enhanced depression care has been called for and entails tailoring treatment to measured outcomes [9].

One way to provide structured treatment guidelines is through algorithms which incorporate various patient characteristics and allocate treatments most likely to be effective. To the authors' knowledge, there is no scientifically evaluated and pragmatic antidepressant decision support tool in UK primary care. This is based on a literature search conducted in September 2019 in the ISRCTN, Eudra CT, Clinical Trials.gov, PubMed and Web of Knowledge databases using the key words: ("depression or depressive") AND "decision" AND ("computer or algorithm") AND "antidepressant". The search returned four relevant studies which employed a computer-based algorithm to help make decisions about antidepressant treatments [11-14].

Out of three completed studies, two found evidence for the effectiveness of algorithm support compared to treatment-as-usual. Adli et al. (2017) [13] found shorter time to remission and fewer medication switches using an algorithm compared to computerised support and treatment-as-usual for depression treatment. Kurian et al. (2009) [11] assigned private GPs and

For peer review only - http://bmjopen.bmj.com/site/about/guidelines.xhtml

their MDD patients to active computerised decision support for antidepressants based on the 1999 Texas Medication Algorithm Project (TMAP) [15] and treatment-as-usual. The active group showed a 41% response rate to treatment vs. 26% in the treatment-as-usual group. However, Rollman et al. (2002) [12] reported no difference in depressive symptoms after six months between active decision-support, non-specific prompting that depression needs treatment and treatment-as-usual. The ongoing Assurex Health Inc study [14] uses a genetic testing decision support tool to predict differential treatment response to treatments for depression.

Overall, all of the above studies were conducted outside of the UK, often in private healthcare settings which are difficult to compare with the UK NHS. Several of the support tools did not provide specific treatment sequences, only broad recommendations to change medication. Furthermore, some of the studies could not translate recommendations into algorithms to be used by GPs so that researchers had to manually provide GPs with advice reports. This creates a complicated treatment process and it seems unlikely that the support tools could provide ongoing recommendations to prescribers after initial treatment recommendations. Moreover, Kurian et al. (2009) [11] and Rollman et al. (2002) [12] employed algorithms that are now outdated and lacked integration of recent evidence on superiority of specific novel antidepressants in terms of tolerability and efficacy. All of the tools lacked integration into existing electronic healthcare record systems that GPs are familiar with. The NICE guidelines provide useful MDD treatment advice for GPs but a scarcity of studies on complex interventions using sequenced treatment algorithms has meant that the guidelines are unclear about how many switches between individual antidepressants are recommended before seeking specialist advice on other strategies i.e. augmentation. The seminal STAR*D study investigating such a sequenced treatment algorithm has used augmentation strategies that according to NICE should not be carried out by GPs [16].

Therefore, the aim of the current study is to assess the feasibilty of a future definitive randomised controlled trial to test the efficacy and cost-effectiveness of a decision tool incorporating an algorithm to advise GPs on antidepressant prescribing for patients who have not responded to first-line treatments. In contrast to earlier work, the treatment algorithm is based on NICE guidelines including the latest health technology appraisals. If successful, the present study will therefore enable the first investigation into whether a sequenced treatment algorithm that relies on well-tolerated single antidepressants is effective and cost-effective. The

For peer review only - http://bmjopen.bmj.com/site/about/guidelines.xhtml

authors have gained support from one of the leading UK providers of primary care electronic record systems (EMIS group) who have implemented the algorithm into their software.

Study Objectives

1) To develop the first computerised decision support tool for antidepressant treatment in UK primary care.

2) To assess the feasibility of a future confirmatory clinical trial by:

    a. estimating lost to follow-up rates;

    b. estimating GP adherence to the algorithm and patient adherence to prescribed medications;

    c. determining the number of GP practices willing to recruit patients for the study (determined by the Clinical Research Network (CRN) who will approach all practices in the participating Clinical Comissioning Groups (CCG));

    d. estimating participant recruitment rates per GP;

    e. estimating GP satisfaction with the decision tool.

3) To provide initial standard deviation estimates and intra-class correlation coefficients for computing effect size estimates for a larger confirmatory trial.

4) To collect health economic estimates of the roll-out cost of the intervention and changes in service use associated with the tool, including psychiatric referrals to mental health teams and/or the study psychiatrist.

Trial Design

The study design is a single-blinded cluster-randomised controlled clinical trial, where the GP practice is the cluster/randomisation unit. Considering complex intervention guidance [17], the study is designed as a feasibility study of the decision support tool to provide estimates of the unknown variables needed to plan a subsequent larger trial.

After being enrolled, patients receive access to a secure mobile phone app developed in the study. Participants enter weekly Maudsley Modified Patient Health Questionnaire (MM-PHQ-9) ratings [18], hypomanic symptoms, Frequency, Intensity and Burden of Side Effects Ratings (FIBSER) medication side effects [19], medication changes and self-blame-related action

For peer review only

tendencies such as hiding. A daily question will ask about medication adherence. Alternatively, participants can opt to provide this data at weekly intervals via post or phone.

Methods

Study Setting

The study is based in UK primary care, recruiting GPs and patients from South London primary care practices. Patients will be assessed at the Institute of Psychiatry, Psychology and Neuroscience in South London and treated at their usual GP practice.

Eligibility criteria for patients

Inclusion criteria: age ≥ 18; at least moderately severe major depressive syndrome on Patient Health Questionnaire (PHQ-9; score≥15) [20]; no plans to change GP practice; able to complete self-report scales orally or in writing; no previous prescription of mirtazapine or vortioxetine; and early treatment resistance. The latter is defined as (i) current or recent prescription (in previous 2 months) of any of the following antidepressants: citalopram, fluoxetine, sertraline, escitalopram, paroxetine, venlafaxine, or duloxetine; and (ii) previous prescription of at least one other antidepressant from the same list.

Exclusion criteria: inability to consent to the study; unstable medical condition; currently being treated by mental health specialist; high suicide risk (Mini International Neuropsychiatric Interview (MINI suicidality screen)) [21]; past diagnosis of schizophrenia or schizo-affective disorder; current psychotic symptoms (three clinical screening questions to exclude schizophreniform disorders [22; 23]); bipolar disorder (WHO Composite International Diagnostic Interview (CIDI) screening scale for bipolar disorder [24]); currently at risk of being violent; drug or alcohol abuse over the last six months (Primary Care Evaluation of Mental Disorders (PRIME-MD [25]), modified to screen for drug abuse); breastfeeding/ within six months of giving birth; and both escitalopram and sertraline have already been prescribed.

Interventions

Participants meeting the eligibility criteria will participate in the intervention arm to which their GP practice is randomised:

Arm 1: Treatment-as-usual (TAU). Treatment as GPs would usually deliver with no decision support and no constraints (these GPs will not be aware of the rules

For peer review only - http://bmjopen.bmj.com/site/about/guidelines.xhtml

of the treatment algorithm used in the other group nor will they be able to access it);

Arm 2: Decision Support Tool. Treatment using the decision support tool to assist GPs with antidepressant prescriptions, prompting GPs to review patients' medications and change them if ineffective. The algorithm and technical requirements of the tool are described below.

An EMIS decision support module was developed which provides suggestions for antidepressant prescribing after highlighting the importance of non-pharmacological strategies such as psychological therapy or exercise. The treatment recommendations are based on NICE guidelines, British Association for Psychopharmacology guidelines [26] and Maudsley prescribing guidelines [27]. The algorithm is designed to incorporate patient preferences, ensure maximum prescribing safety and offer guidance on antidepressant monotherapy (avoiding combinations). The NICE guidelines state that GPs should not normally initiate combinations without consulting a psychiatrist first [7] (for the rationale and details of the tool recommendations see Supplementary Methods).

The module is implemented only for those GP practices taking part in the study and only for participating patients at those practices. GPs will use the EMIS module during patient appointments by entering patients' MM-PHQ-9 scores [18] of their depressive symptoms over the last week from the study mobile app. The tool will offer treatment advice on whether a patient's antidepressant should be maintained or changed and what it should be changed to. The advisor tool is used for every depression review appointment over the 14-week study duration, with no minimum number of appointments.

GPs in both conditions will receive training by the study Principal Investigator (PI) on study procedures including adverse event reporting and incorporating patients' mobile app data. Training will be tailored to treatment arm so that GPs in the decision support tool arm will be trained in how to use the EMIS module to guide treatment decisions whereas GPs in the TAU arm will be advised to use their clinical judgement as usual for treatment.

Adherence

Adherence to the prescribed drugs in both arms will be monitored using the available tools on EMIS as well as the mobile app. Patients will be withdrawn from the antidepressant early in the event of a serious or medically important adverse event considered by the GP to be related to the intervention, hypomania, or a significant deterioration of symptoms requiring referral to

For peer review only

secondary care. GP adherence to the algorithm will be measured after GPs have completed their participation in the study with a self-report questionnaire and information from EMIS.

Outcomes

Feasibility outcome measures:

1) Lost to follow-up rates;
2) GP adherence to the algorithm for each completed patient rated by a trial clinician (0 – 3 for none, less than 50%, 50% or more or 100% of recommended steps implemented);
3) Average patient adherence to prescribed medications based on EMIS electronic prescribing records;
4) Adverse Event (AE) and Serious Adverse Event (SAE) rates (grade and relationship to intervention);
5) Patient adherence to GP attendance measured by % of attended GP visits out of scheduled visits on EMIS over treatment period;
6) Recruitment rates;
7) Average GP satisfaction with decision support tool (intervention arm; after GP completion of study);
8) DSM-IV Social and Occupational Functioning Assessment Scale (SOFAS) scale of psychosocial functioning (on final visit, whilst modelling baseline score) [28];
9) Maudsley Visual Analogue Mood Scale (on final visit, whilst modelling baseline score);

Primary clinical outcome measure:

10) Self-rated Quick Inventory of Depressive Symptomatology sum score (QIDS-SR16) [29] at final visit, adjusting for baseline score;

Secondary clinical outcome measures:

11) Depressive symptoms assessed by the Montgomery–Asberg Depression Rating Scale (MADRS) at follow-up assessment, adjusting for baseline score [30];
12) Clinical Global Impression scale; change between baseline and follow-up assessment (final visit) [31];
13) Generalised Anxiety Disorder (GAD-7) scale at follow-up assessment, adjusting for baseline score [25];
14) Body Mass Index at follow-up assessment adjusting for baseline score;

For peer review only - http://bmjopen.bmj.com/site/about/guidelines.xhtml

Exploratory clinical outcome measures:

15) Average score for medication side effects on FIBSER [19]; self-report via mobile app;

16) Average % of adherence to prescribed antidepressant medication (self-report via mobile app);

17) Average MM-PHQ-9 scores in last 2 weeks (at follow-up, whilst modelling first 2 weeks as baseline average) [18];

Health Economics measures:

18) Service Use as determined on EMIS including psychiatric referrals and referrals to study psychiatrist, as well as time to psychiatric referral; also primary care consultation rates;

19) Service Use; self-reported using a modified version of the Adult Service Use Schedule (AD-SUS) [32];

20) Quality of life using the EQ-5D-3L [33] - the standard measure recommended by NICE for cost-effectiveness analyses.

Participant timeline

Interested participants will complete an online pre-screening assessment, followed by a face-to-face assessment including a clinical evaluation to determine full eligibility. The results of the screening assessment will be shared with GPs and patients. Inclusion decisions will not be made by GPs to help mitigate against selection biases and will be decided by the research team after the screening assessment. Eligible patients will undergo treatment for depression over 14 weeks with their GPs. In both arms, patients will be seen by their GP who will review and monitor treatment. Patients' participation will last for approximately 15-18 weeks after their face-to-face assessment, at which point their follow-up assessment will be conducted. See Table 1 below for participant timeline.

For peer review only

Table 1- Participant Timeline

	Participants identified by EMIS search tool	Consent for contact	Initial Pre-screening (electronic, letter, or phone)	Pre-Trial Assessment in person	Treatment Sessions arranged by GP as soon as possible after pre-trial assessment with no fixed number over 14 weeks	Post-Trial assessment in person after 15-18 weeks since Pre-Trial assessment
Participants contacted about study	X					
Reply by letter slip, phone, text or email		X				
Oral/electronic or written pre-screening informed consent			X			
Introduction to the study			X			
Assessment of eligibility			X	X		
Written informed consent				X		
Clinical assessment & neuro-psychological tests				X		X
Trial intervention delivered by GPs					X	
Mobile app/ phone weekly assessment					X	

Sample size

Each practice will be asked to enrol approximately 8-11 participants. The study aims to enrol 86 participants assuming the same lost to follow-up rate as in Kurian et al.'s (2009) study [11](18%), giving a final sample size of 70 (35 in each group as recommended [34]). This will enable estimation of the lost to follow-up rate within a 95% confidence interval of ± 8% [35]. Kurian et al. [11] did not provide effect size estimates so the present study has been designed to provide means and standard deviations, as well as confidence intervals for measures of

For peer review only - http://bmjopen.bmj.com/site/about/guidelines.xhtml

change on the primary outcome measure (QIDS-SR16 [29]) as recommended for feasibility trials [34].

Recruitment of GP Practices

The study aims to recruit 8-20 GP practices. Only one GP per practice will be recruited to the study to avoid communication about treatment allocation between GPs. In the event of a GP leaving a participating practice, an alternative GP from the same practice will replace them.

Enrolment of Participants

Patients will be enrolled after GPs are randomised. An EMIS eligibility tool developed for the study will identify potential participants from each practice meeting the inclusion/exclusion criteria (see the Supplementary Methods for further details). Practice/CRN staff will run the EMIS search and send eligible patients a study advert and request for consent for contact via post.

Assignment of Interventions

Allocation

Sequence Generation: The randomisation service will be provided by the King's Clinical Trials Unit (KCTU) in accordance with a standard operating procedure and held on a secure server. GP practices will be randomised in pairs with a 1:1 allocation to either intervention or TAU using block randomisation with random permuted blocks of block size two. This will help maintain allocation concealment while ensuring a similar number of practices are allocated to each arm.

Concealment Mechanism: KCTU will send the randomisation outcome to unblinded researchers only to ensure researchers conducting assessments remain blind. The randomisation details will be kept on a password-protected network drive only accessible by the PI.

Blinding

Patients, researchers assessing observer-rated outcomes and the senior statistician will be blind to treatment allocation. All other members of the study team, including the junior statistician, and GPs will be unblinded. Unblinding incidents will be recorded in the trial master file and reported to the Trial Steering Committee (TSC; see Data Monitoring section). Unblinded assessments will be retained in the study. If a researcher becomes unblinded prior to completing

For peer review only - http://bmjopen.bmj.com/site/about/guidelines.xhtml

assessments, an alternative researcher will conduct future assessments with patients from that practice in order to conduct unbiased assessments. There is no requirement for an emergency unblinding procedure, as the PI and GPs are not blinded.

Data collection Methods

The pre-screening assessment is conducted online using a survey software and includes the PRIME-MD (self-report) [25] to assess depressive symptoms and alcohol abuse and modified to screen for drug abuse, the CIDI (self-report) for bipolar disorder [24], questions to exclude schizophreniform disorders and psychotic depression [22; 23], and pregnancy in women. Eligible participants are invited to a face-to-face assessment which includes depression history, treatment history, medical history, MINI suicidality screen [21], DSM-5 Structured Clinical Diagnostic Interview [28], Psychiatric Family History Screen [36], AMDP Psychopathology Interview questions of depression items [37; 38], Addenbrooke's Cognitive Examination-III in patients over 50 to detect early Alzheimer's disease [39], Longitudinal Interval Follow-up Interview (LIFE) [40], and the Young Mania Rating Scale (YMRS) [41]. Participants' medical records will be checked to confirm medical details. Participants will use the mobile app for the 14-week study duration.

At the follow-up assessment, the study outcome measures and YMRS [41] will be repeated. The LIFE [40] will be used to determine remission and its psychiatric status rating scale used as a comparison to baseline. All assessments will be conducted by trained researchers. The research team will be trained and closely supervised, establishing sufficient inter-rater reliability on semi-structured interviews with the PI before conducting assessments independently [23].

Patient and Public Involvement

The study is supported by a service user advisory group which provides input to the study. This group meets on a regular basis and provides insight into the study design, information disseminated to patients and the burden of trial participation from the patient's perspective. At the end of the study, the service user advisory group will comment on the findings and contribute to the dissemination plan.

For peer review only

Statistical Methods

Outcomes

Categorical outcomes (e.g. loss to follow-up) will be described using frequencies and proportions. The QIDS-SR16 and other continuous outcomes will be summarised at baseline and follow-up using means and standard deviations. The GP practice intra-class correlation will be calculated for the outcome variable using one-way random effects analysis of covariance (adjusted for baseline). A preliminary analysis of the difference between the groups, as far as possible using the intention-to-treat principle, is planned. This analysis will be identified as preliminary and underpowered when published, and no *p* values will be provided. Continuous outcomes measured at baseline and follow-up only, such as the QIDS-SR16, will be analysed using linear regression with robust standard error calculation to account for clustering within GP practice. Continuous outcomes measured weekly will be analysed using mixed linear regression models with a random intercept for GP practice to account for clustering. Both sets of models will include treatment arm as a covariate and will adjust for baseline measure of the outcomes where appropriate. Any missing baseline data will be imputed using mean imputation [42]. Missing data in weekly outcomes will be accounted for under the missing at random assumption by using the maximum likelihood algorithm to fit the mixed models. We may consider multiple imputation for outcomes measured only at follow-up if post-randomisation variables can be quantified and are related to having missing outcomes. Adverse events (see Harms section) will be summarised separately as AEs and SAEs, by intervention group, as number of events and number of people experiencing events.

Data Monitoring

The TSC will meet bi-annually to oversee study progress and comprises the study PI, research associate, research assistants, statisticians, collaborators and service users. There are no formal criteria for terminating the trial. Informal decisions about early trial termination will be made by the TSC and sponsor should they have major concerns about safety or conduct of the trial. This would be considered in the event that extensive recurrent serious or medically important adverse events are observed, particularly if these are Suspected Unexpected Serious Adverse Reactions (SUSARs).

Harms

GPs participating in the study, especially in the intervention arm, are likely to change medications more often and prescribe medications which are less frequently used in primary

For peer review only

care. All medications recommended by the decision support tool are in accordance with NICE guidance and were carefully selected for the study based on likelihood of positive effects and side effects (see Supplementary Methods). Kurian et al. (2009) [11] found their support tool to be beneficial for depression treatment, although some patients felt worse as a result of changing medications – for example, having more suicidal thoughts. Such side effects will be monitored by GPs and discussed with patients.

AEs/SAEs are defined in the Supplementary Methods. AEs/SAEs will be recorded from the time the subject provides informed consent at the baseline assessment until their follow-up. GPs will record all AEs/SAEs on EMIS and they will be regularly collected from GPs by the research team and assessed. AEs/SAEs reported to the PI will be recorded on AE forms as part of the case report form using an ID number to identify the patient. AEs/SAEs will be reported bi-annually to the TSC and an unblinded sub-committee, assuming the functions of the data monitoring committee, will review the information separated by arm.

For peer review only

Ethics and Dissemination

Research Ethics Approval

The study has received NHS ethical approval from the London - Camberwell St Giles Research Ethics Committee (ref:17/LO/2074).

Protocol Amendments

Modifications to the protocol will be conveyed to the TSC at the planning stage for members to provide input, then again to inform the committee of the ethics committee decision.

Consent

Participants will provide informed consent to take part in the study and are free to withdraw at any time with no reason given (see Supplementary Methods for further detail).

Dissemination

The trial has been pre-registered on ClinicalTrials.gov (Identifier: NCT03628027). See Supplementary Methods for further detail on dissemination.

Health economics

For peer review only - http://bmjopen.bmj.com/site/about/guidelines.xhtml

The study will not include a formal economic evaluation, instead the focus is on developing the tools needed to support a future trial. First, a meaningful unit cost will be calculated for our tool in collaboration with EMIS. The unit cost will consider the long-term roll-out cost of the intervention and investigate how to allocate this to an individual patient in order to carry out a future cost-effectiveness analysis. Second, the methods for the collection of service use data will be optimised, which will include a comparison of data collected from EMIS and data collected from patients in self-report.

Discussion

The study is the first randomised trial to implement a computerised decision-support tool into UK primary care to aid GPs with antidepressant prescribing. The tool uses up-to-date NICE guidelines to provide step-by-step guidance on decisions to increase, switch or cease antidepressants.

Implications

The Antidepressant Advisor tool is designed to be easy to use and to save time for GPs who are often limited to 10-minute appointments. The tool could ensure good quality and consistent antidepressant care in UK primary care despite little time or resources. Therefore, if the trial is found to be feasible, the aim is to apply for funding for a definitive trial.

Conclusion

The Antidepressant Advisor tool is a computerised decision-support tool which implements an algorithm for allocating antidepressants to those who have previously not responded to treatment. The tool is currently being tested as part of a feasibility trial in order to understand whether its implementation into the existing NHS primary care system will be acceptable and useful for both GPs and patients.

For peer review only

References

1. WHO. The global burden of disease: 2004 Update. 2008.
2. UK National Centre for Social Research. Adult Psychiatric Morbidity Survey. 2007.
3. Dormon F. Is mental health care improving?: The Health Foundation. 2015.
4. Kessler RC, Berglund P, Demler O et al. . The epidemiology of major depressive disorder: results from the National Comorbidity Survey Replication (NCS-R). JAMA 2003;289(23):3095-105.
5. Trivedi MH, Rush AJ, Wisniewski SR et al. . Evaluation of outcomes with citalopram for depression using measurement-based care in STAR*D: implications for clinical practice. Am J Psychiatry 2006;163(1):28-40.
6. Gyani A, Shafran R, Layard R, Clark DM. Enhancing recovery rates: lessons from year one of IAPT. Behav Res Ther 2013;51(9):597-606.
7. NICE. Depression: The treatment and management of depression in adults; 2010. City: The British Psychological Society and The Royal College of Psychiatrists.
8. Symons L, Tylee A, Mann A et al. . Improving access to depression care: descriptive report of a multidisciplinary primary care pilot service. Br J Gen Pract 2004;54(506):679-83.
9. Von Korff M, Goldberg D. Improving outcomes in depression. BMJ 2001;323(7319):948-9.
10. Spence R, Roberts A, Ariti C, Bardsley M. Focus On: Antidepressant prescribing. The Health Foundation & Nuffield Trust; 2014.
11. Kurian BT, Trivedi MH, Grannemann BD et al. . A computerized decision support system for depression in primary care. Prim Care Companion J Clin Psychiatry 2009;11(4):140-6.
12. Rollman BL, Hanusa BH, Lowe HJ et al. . A randomized trial using computerized decision support to improve treatment of major depression in primary care. J Gen Intern Med 2002;17(7):493-503.
13. Adli M, Wiethoff K, Baghai TC et al. . How effective is algorithm-guided treatment for depressed inpatients? Results from the randomized controlled multicenter German Algorithm Project 3 trial. International Journal of Neuropsychopharmacology 2017;20(9):721-730.
14. Inc AH. Pharmacogenomic Decision Support With GeneSight Psychotropic to Guide the Treatment of Major Depressive Disorder. ClinicalTrials.gov registered 2015.
15. Crismon ML, Trivedi M, Pigott TA et al. . The Texas Medication Algorithm Project: report of the Texas Consensus Conference Panel on Medication Treatment of Major Depressive Disorder. J Clin Psychiatry 1999;60(3):142-56.
16. Rush AJ, Fava M, Wisniewski SR et al. . Sequenced treatment alternatives to relieve depression (STAR* D): rationale and design. Controlled clinical trials 2004;25(1):119-142.
17. MRC. Developing and evaluating complex interventions: new guidance. 2006.
18. Walton S. Developing a novel outcome measure for depression: The Maudsley Modified Patient Health Questionnaire-9. . Unpublished Masters dissertation, King's College London. 2019.
19. Wisniewski SR, Rush AJ, Balasubramani GK et al. . Self-rated global measure of the frequency, intensity, and burden of side effects. J Psychiatr Pract 2006;12(2):71-9.
20. Spitzer RL, Kroenke K, Williams JW, and the Patient Health Questionnaire Primary Care Study G. Validation and utility of a self-report version of prime-md: The phq primary care study. JAMA 1999;282(18):1737-1744.
21. Sheehan DV, Lecrubier Y, Sheehan KH et al. . The Mini-International Neuropsychiatric Interview (M.I.N.I.): the development and validation of a structured diagnostic

For peer review only

psychiatric interview for DSM-IV and ICD-10. J Clin Psychiatry 1998;59 Suppl 20:22-33;quiz 34-57.
22. Green MF, Hellemann G, Horan WP et al. . From perception to functional outcome in schizophrenia: modeling the role of ability and motivation. Archives of General Psychiatry 2012;69(12):1216-1224.
23. Lythe KE, Moll J, Gethin JA et al. . Self-blame–selective hyperconnectivity between anterior temporal and subgenual cortices and prediction of recurrent depressive episodes. JAMA psychiatry 2015;72(11):1119-1126.
24. Kessler RC, Akiskal HS, Angst J et al. . Validity of the Assessment of Bipolar Spectrum Disorders in the WHO CIDI 3.0. Journal of affective disorders 2006;96(3):259-269.
25. Spitzer RL, Williams JW, Kroenke K, et al. Utility of a new procedure for diagnosing mental disorders in primary care: The prime-md 1000 study. JAMA 1994;272(22):1749-1756.
26. Cleare A, Pariante CM, Young AH et al. . Evidence-based guidelines for treating depressive disorders with antidepressants: A revision of the 2008 British Association for Psychopharmacology guidelines. J Psychopharmacol 2015;29(5):459-525.
27. Taylor D, Paton C, Kapur S, South London and Maudsley NHS Trust. The Maudsley prescribing guidelines in psychiatry: Wiley. 2015.
28. First MB SR, Gibbon M, Williams JBW. Structured Clinical Interview for DSM-IV-TR Axis I Disorders, Research Version, Patient Edition. (SCID-I/P). . New York, NY: Biometrics Research, New York State Psychiatric Institute 2002.
29. Rush AJ, Trivedi MH, Ibrahim HM et al. . The 16-Item Quick Inventory of Depressive Symptomatology (QIDS), clinician rating (QIDS-C), and self-report (QIDS-SR): a psychometric evaluation in patients with chronic major depression. Biol Psychiatry 2003;54(5):573-83.
30. Montgomery SA, Åsberg M. A new depression scale designed to be sensitive to change. British Journal of Psychiatry 1979;134(4):382-389.
31. Busner J, Targum SD, Miller DS. The Clinical Global Impressions scale: errors in understanding and use. Compr Psychiatry 2009;50(3):257-62.
32. Kuyken W HR, Barrett B, BynG R, Dalgleish T, Kessler D et al. Effectiveness and cost-effectiveness of mindfulness-based cognitive therapy compared with maintenance antidepressant treatment in the prevention of depressive relapse or recurrence (PREVENT): a randomised controlled trial. . The Lancet. 2015;386:63-73.
33. R. B. EuroQol: the current state of play. Health Policy. 1996.;37:53-72.
34. Teare MD, Dimairo M, Shephard N et al. . Sample size requirements to estimate key design parameters from external pilot randomised controlled trials: a simulation study. Trials 2014;15:264.
35. Lwanga SK, Lemeshow S, Organization WH. Sample size determination in health studies: a practical manual. 1991.
36. Weissman MM WP, Adams P, Wolk S, Verdeli H, Olfson M. . Brief screening for family psychiatric history: the family history screen. . Arch Gen Psychiatry 2000;57:675.
37. Fähndrich E RE. The AMDP system for the documentation of psychiatric symptoms: course and effectivity of a training seminar. Pharmacopsychiatry 1985;18:278-281.
38. Fähndrich E SR. Leitfaden zur Erfassung des psychopathologischen Befundes: halbstrukturiertes Interview anhand des AMDP-Systems. Hogrefe Verlag 1989.
39. Noone P. Addenbrooke's cognitive examination-III. Occupational Medicine 2015;65(5):418-420.
40. Keller MB, Lavori PW, Friedman B et al. . The Longitudinal Interval Follow-up Evaluation. A comprehensive method for assessing outcome in prospective longitudinal studies. Arch Gen Psychiatry 1987;44(6):540-8.

For peer review only - http://bmjopen.bmj.com/site/about/guidelines.xhtml

41. Young RC, Biggs JT, Ziegler VE, Meyer DA. A rating scale for mania: reliability, validity and sensitivity. Br J Psychiatry 1978;133:429-35.
42. White IR, Thompson SG. Adjusting for partially missing baseline measurements in randomized trials. Stat Med 2005;24(7):993-1007.

For peer review only

STANDARD PROTOCOL ITEMS: RECOMMENDATIONS FOR INTERVENTIONAL TRIALS

SPIRIT 2013 Checklist: Recommended items to address in a clinical trial protocol and related documents*

Section/item	Item No	Description
**Administrative information**		
Title	1	Descriptive title identifying the study design, population, interventions, and, if applicable, trial acronym
Trial registration	2a	Trial identifier and registry name. If not yet registered, name of intended registry
	2b	All items from the World Health Organization Trial Registration Data Set
Protocol version	3	Date and version identifier
Funding	4	Sources and types of financial, material, and other support
Roles and responsibilities	5a	Names, affiliations, and roles of protocol contributors
	5b	Name and contact information for the trial sponsor
	5c	Role of study sponsor and funders, if any, in study design; collection, management, analysis, and interpretation of data; writing of the report; and the decision to submit the report for publication, including whether they will have ultimate authority over any of these activities
	5d	Composition, roles, and responsibilities of the coordinating centre, steering committee, endpoint adjudication committee, data management team, and other individuals or groups overseeing the trial, if applicable (see Item 21a for data monitoring committee)
**Introduction**		
Background and rationale	6a	Description of research question and justification for undertaking the trial, including summary of relevant studies (published and unpublished) examining benefits and harms for each intervention
	6b	Explanation for choice of comparators
Objectives	7	Specific objectives or hypotheses
Trial design	8	Description of trial design including type of trial (eg, parallel group, crossover, factorial, single group), allocation ratio, and framework (eg, superiority, equivalence, noninferiority, exploratory)

For peer review only

Methods: Participants, interventions, and outcomes

Study setting	9	Description of study settings (eg, community clinic, academic hospital) and list of countries where data will be collected. Reference to where list of study sites can be obtained
Eligibility criteria	10	Inclusion and exclusion criteria for participants. If applicable, eligibility criteria for study centres and individuals who will perform the interventions (eg, surgeons, psychotherapists)
Interventions	11a	Interventions for each group with sufficient detail to allow replication, including how and when they will be administered
	11b	Criteria for discontinuing or modifying allocated interventions for a given trial participant (eg, drug dose change in response to harms, participant request, or improving/worsening disease)
	11c	Strategies to improve adherence to intervention protocols, and any procedures for monitoring adherence (eg, drug tablet return, laboratory tests)
	11d	Relevant concomitant care and interventions that are permitted or prohibited during the trial
Outcomes	12	Primary, secondary, and other outcomes, including the specific measurement variable (eg, systolic blood pressure), analysis metric (eg, change from baseline, final value, time to event), method of aggregation (eg, median, proportion), and time point for each outcome. Explanation of the clinical relevance of chosen efficacy and harm outcomes is strongly recommended
Participant timeline	13	Time schedule of enrolment, interventions (including any run-ins and washouts), assessments, and visits for participants. A schematic diagram is highly recommended (see Figure)
Sample size	14	Estimated number of participants needed to achieve study objectives and how it was determined, including clinical and statistical assumptions supporting any sample size calculations
Recruitment	15	Strategies for achieving adequate participant enrolment to reach target sample size

Methods: Assignment of interventions (for controlled trials)

Allocation:

Sequence generation	16a	Method of generating the allocation sequence (eg, computer-generated random numbers), and list of any factors for stratification. To reduce predictability of a random sequence, details of any planned restriction (eg, blocking) should be provided in a separate document that is unavailable to those who enrol participants or assign interventions

For peer review only - http://bmjopen.bmj.com/site/about/guidelines.xhtml

Allocation concealment mechanism	16b	Mechanism of implementing the allocation sequence (eg, central telephone; sequentially numbered, opaque, sealed envelopes), describing any steps to conceal the sequence until interventions are assigned
Implementation	16c	Who will generate the allocation sequence, who will enrol participants, and who will assign participants to interventions
Blinding (masking)	17a	Who will be blinded after assignment to interventions (eg, trial participants, care providers, outcome assessors, data analysts), and how
	17b	If blinded, circumstances under which unblinding is permissible, and procedure for revealing a participant’s allocated intervention during the trial
**Methods: Data collection, management, and analysis**		
Data collection methods	18a	Plans for assessment and collection of outcome, baseline, and other trial data, including any related processes to promote data quality (eg, duplicate measurements, training of assessors) and a description of study instruments (eg, questionnaires, laboratory tests) along with their reliability and validity, if known. Reference to where data collection forms can be found, if not in the protocol
	18b	Plans to promote participant retention and complete follow-up, including list of any outcome data to be collected for participants who discontinue or deviate from intervention protocols
Data management	19	Plans for data entry, coding, security, and storage, including any related processes to promote data quality (eg, double data entry; range checks for data values). Reference to where details of data management procedures can be found, if not in the protocol
Statistical methods	20a	Statistical methods for analysing primary and secondary outcomes. Reference to where other details of the statistical analysis plan can be found, if not in the protocol
	20b	Methods for any additional analyses (eg, subgroup and adjusted analyses)
	20c	Definition of analysis population relating to protocol non-adherence (eg, as randomised analysis), and any statistical methods to handle missing data (eg, multiple imputation)
**Methods: Monitoring**		
Data monitoring	21a	Composition of data monitoring committee (DMC); summary of its role and reporting structure; statement of whether it is independent from the sponsor and competing interests; and reference to where further details about its charter can be found, if not in the protocol. Alternatively, an explanation of why a DMC is not needed

For peer review only - http://bmjopen.bmj.com/site/about/guidelines.xhtml

	21b	Description of any interim analyses and stopping guidelines, including who will have access to these interim results and make the final decision to terminate the trial
Harms	22	Plans for collecting, assessing, reporting, and managing solicited and spontaneously reported adverse events and other unintended effects of trial interventions or trial conduct
Auditing	23	Frequency and procedures for auditing trial conduct, if any, and whether the process will be independent from investigators and the sponsor
**Ethics and dissemination**		
Research ethics approval	24	Plans for seeking research ethics committee/institutional review board (REC/IRB) approval
Protocol amendments	25	Plans for communicating important protocol modifications (eg, changes to eligibility criteria, outcomes, analyses) to relevant parties (eg, investigators, REC/IRBs, trial participants, trial registries, journals, regulators)
Consent or assent	26a	Who will obtain informed consent or assent from potential trial participants or authorised surrogates, and how (see Item 32)
	26b	Additional consent provisions for collection and use of participant data and biological specimens in ancillary studies, if applicable
Confidentiality	27	How personal information about potential and enrolled participants will be collected, shared, and maintained in order to protect confidentiality before, during, and after the trial
Declaration of interests	28	Financial and other competing interests for principal investigators for the overall trial and each study site
Access to data	29	Statement of who will have access to the final trial dataset, and disclosure of contractual agreements that limit such access for investigators
Ancillary and post-trial care	30	Provisions, if any, for ancillary and post-trial care, and for compensation to those who suffer harm from trial participation
Dissemination policy	31a	Plans for investigators and sponsor to communicate trial results to participants, healthcare professionals, the public, and other relevant groups (eg, via publication, reporting in results databases, or other data sharing arrangements), including any publication restrictions
	31b	Authorship eligibility guidelines and any intended use of professional writers
	31c	Plans, if any, for granting public access to the full protocol, participant-level dataset, and statistical code

For peer review only

Appendices

Informed consent materials	32	Model consent form and other related documentation given to participants and authorised surrogates
Biological specimens	33	Plans for collection, laboratory evaluation, and storage of biological specimens for genetic or molecular analysis in the current trial and for future use in ancillary studies, if applicable

*It is strongly recommended that this checklist be read in conjunction with the SPIRIT 2013 Explanation & Elaboration for important clarification on the items. Amendments to the protocol should be tracked and dated. The SPIRIT checklist is copyrighted by the SPIRIT Group under the Creative Commons "Attribution-NonCommercial-NoDerivs 3.0 Unported" license.

For peer review only

SUPPLEMENTARY MATERIALS

Study Protocol for The Antidepressant Advisor (ADeSS): A Decision Support System for Antidepressant Treatment for Depression in UK primary care - a feasibility study

Short Title: The Antidepressant Advisor

Supplementary Methods

Software Eligibility Tool Criteria

1) current or recent prescription (in the last 2 months) of any of the following antidepressants listed: citalopram, fluoxetine, sertraline, escitalopram, paroxetine, venlafaxine, or duloxetine
2) previous prescription of at least one other antidepressant out of the same list of antidepressants
3) exclude patients for whom both escitalopram and sertraline have already been prescribed
4) no previous prescription of mirtazapine or vortioxetine
5) no mania/hypomania/bipolar (or synonyms), psychosis/schizophrenia (or synonyms), or drug abuse/misuse/dependence (or synonyms), or alcohol abuse/misuse/dependence or synonyms
6) age≥18 years
7) not pregnant
8) not given birth in last 6 months.

Decision Support Tool Rationale & Details

The decision chart for the decision support tool is presented in Figure 1 below. A network meta-analysis, not including vortioxetine, showed that sertraline and escitalopram are superior in terms of acceptability and efficacy compared to other antidepressants [1]. Since sertraline is among the first–line options recommended by NICE, if patients tolerate serotonin reuptake inhibitors (SSRI), the algorithm starts with recommending escitalopram for those patients where two NICE recommended first-line treatments have failed including sertraline, but starts with sertraline should it not have been prescribed before. For patients who have not tolerated SSRIs (verified by GP), the algorithm makes alternative suggestions. Mirtazapine is among the most efficacious antidepressants [1] and has a different side effect profile to SSRIs which is why it is suggested for these patients. Because of its 75% risk in inducing weight

For peer review only - http://bmjopen.bmj.com/site/about/guidelines.xhtml

gain and thereby increasing the risk of metabolic syndrome[2], the algorithm suggests vortioxetine in those patients who are at an increased risk of metabolic syndrome (Body Mass Index(BMI)>25, HbA1c increased), because of its low weight-gain-induction risk[3]. Vortioxetine has SSRI properties, but is less likely to induce typical side effects, apart from nausea [3]. A recent NICE health technology appraisal [4]has given a positive recommendation for using vortioxetine in patients whose depressive episode has responded inadequately to two antidepressants. The REVIVE trial [3] showed the benefits of vortioxetine in patients with current MDD who had not responded to SSRI or dual-reuptake inhibitor monotherapy. The appraisal also concluded that vortioxetine may have a better overall safety profile than other antidepressants. For those patients tolerating SSRIs who do not respond to escitalopram and are at an increased metabolic syndrome risk, the algorithm therefore suggests vortioxetine.

Venlafaxine, which is among the most efficacious medications[1], was omitted from the algorithm, because of its higher risk of death in overdose which limits its use in primary care according to the Medicines and Healthcare Regulatory Agency [5]guidance and most GPs we discussed this with are not prepared to prescribe it at the higher doses at which it acts as a noradrenaline reuptake inhibitor. Duloxetine was excluded as an alternative as it was less efficacious in a network meta-analysis [1].

For escitalopram non-responders who are at low metabolic syndrome risk, the algorithm suggests mirtazapine as a next step. For mirtazapine non-responders, a switch to vortioxetine is suggested as a next step. For vortioxetine non-responders during the second or third step, a psychiatric referral to the study psychiatrist is suggested, who could then recommend higher side effect burdened alternatives such as high-dose venlafaxine or augmentation strategies according to NICE guidelines. Agomelatine has been omitted from the algorithm despite its suitability for patients who are not tolerating SSRIs, because there is no positive HTA recommendation for it and because it requires liver enzyme checks [6].

For peer review only - http://bmjopen.bmj.com/site/about/guidelines.xhtml

Figure 1-Decision chart for decision support tool

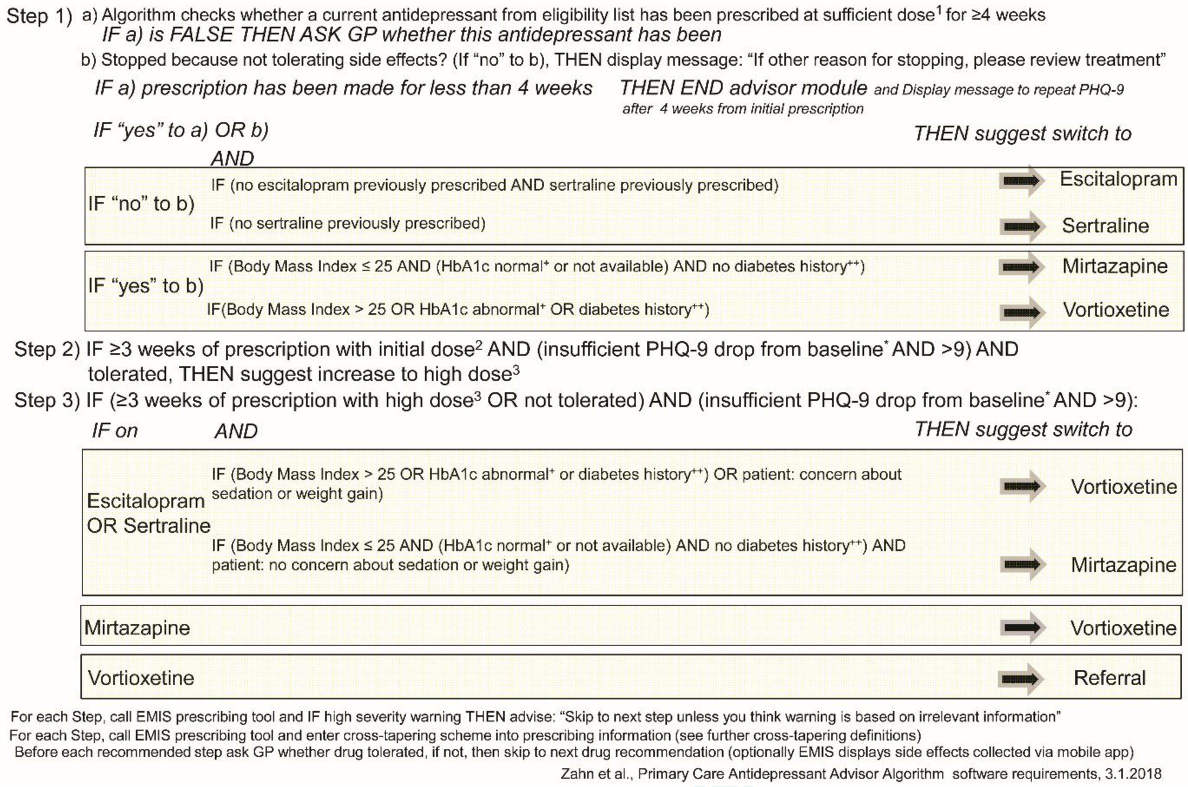

Data Management

Data will be entered electronically into a password-protected database and stored separately from identifiable information. Data validation will be applied to ensure that entered data follows the correct format and is within a valid range. Data will be stored electronically in the master folder and will only be accessed by members of the research team. Data will be backed up by the research associate every time the database is added to on an external hard drive.

Harms

An adverse event (AE) is any untoward medical occurrence in a patient or healthy volunteer, which does not necessarily have a causal relationship with the study procedures. A Serious Adverse Event (SAE) is any undesirable medical occurrence in a participant that is life

For peer review only - http://bmjopen.bmj.com/site/about/guidelines.xhtml

threatening, requires hospitalisation, results in persistent/significant disability/incapacity, causes a congenital anomaly/ birth defect or results in death.

These events may be spontaneously revealed by the subject or observed by the GP and will be recorded regardless of whether the GP believes them to be related to the intervention. The following aspects of AEs/SAEs will be assessed: symptom, possible diagnosis or syndrome, date and duration of AE/SAE, causal relationship with investigation (definite / probable / possible / unlikely / not related), outcome (resolved / resolving / not resolved / resolved with sequelae / fatal / unknown), treatment of event, if applicable (e.g. any medication as well as any other medical measures), actions taken regarding assessment.

GPs will be asked to report SAEs within 24 hours to Lambeth CCG and the PI via email or phone without details which are then followed up with a more detailed report. SAEs which according to the GP are clearly unrelated to the study intervention (e.g. hospitalization for an unrelated illness) and AEs expected when treating depression[i], even if there is a possible relation to the intervention, are exempt from this immediate reporting and will instead be reported together with other AEs at the earliest opportunity (at least quarterly). An executive committee formed of two members of the TSC (one independent) will discuss any AEs/SAEs prior to each TSC then report any concerns back to the TSC. Blinded members of the TSC will be asked to leave the room for the discussion of AEs/SAEs in order to remain blinded. Medically important events or SAEs will be assessed by a medically qualified doctor in the study team and discussed with the TSC. If possible, blinded doctors will assess serious or medically important events, whereas other AEs may be classified by a non-blinded doctor. Classification into SUSAR vs. expected ones will be based on the most updated versions of the summary product characteristics of the prescribed drugs on https://www.medicines.org.uk/emc/. SUSARs would be reported to the sponsor and ethics committee. GPs will be asked to document all other AEs and reasons for withdrawing from the study on EMIS.

Consent

Consent and screening will be carried out in two stages. First, patients identified by the EMIS eligibility tool will be asked to provide written consent to participate in the online pre-screening assessment, with phone and postal options also offered. Second, patients found to be eligible at pre-screening will be invited to the baseline assessment where they will be

For peer review only - http://bmjopen.bmj.com/site/about/guidelines.xhtml

asked to provide written consent to participate in the feasibility trial. Each participant will receive an oral and written explanation of the purposes, procedures, and risks of this study. Written consent will be obtained after explaining the goals, procedures, risks (and discomforts), benefits, and nature of the study to patients. Only adult volunteers and patients who are capable of understanding the study procedures will be allowed to sign the consent forms.

Participants can withdraw from the trial at any time and this will be made clear in the information sheets and consent forms. The reason for withdrawal does not need to be provided.

Dissemination Policy

Reporting bias will be avoided by a priori registration in a public trial register in addition to publication of the current trial protocol as the research is ongoing. All members of the TSC will have the opportunity to review and have input into the current protocol. The results of the study will be published in a pre-publication archive within one year after completion.

For peer review only

References

1. Cipriani A, Furukawa TA, Salanti G et al. Comparative efficacy and acceptability of 12 new-generation antidepressants: a multiple-treatments meta-analysis. Lancet 2009;373(9665):746-58.
2. Masand PS, Gupta S. Long-term side effects of newer-generation antidepressants: SSRIS, venlafaxine, nefazodone, bupropion, and mirtazapine. Ann Clin Psychiatry 2002;14(3):175-82.
3. Montgomery SA, Nielsen RZ, Poulsen LH, Häggström L. A randomised, double-blind study in adults with major depressive disorder with an inadequate response to a single course of selective serotonin reuptake inhibitor or serotonin–noradrenaline reuptake inhibitor treatment switched to vortioxetine or agomelatine. Human Psychopharmacology: Clinical and Experimental 2014;29(5):470-482.
4. NICE. Vortioxetine for treating major depressive episodes - Technology appraisal guidance. 2015.
5. (MHRA). MaHRA. Selective serotonin reuptake inhibitors (SSRIs) and serotonin and noradrenaline reuptake inhibitors (SNRIs): use and safety. . 2014.
6. Agomelatine (Valdoxan): risk of liver toxicity. . Medicines and Healthcare Regulatory Agency (MHRA). 2014.

---

[i] These include non-fatal suicide attempt, non-suicidal self-harm-related injury, non-fatal traffic accident under medication influence, treatment-emergent [hypo-]mania with associated non-fatal complications, non-fatal somatic or anxiety-related complaints leading to A&E visits).

For peer review only - http://bmjopen.bmj.com/site/about/guidelines.xhtml

BMJ Open

Study Protocol for The Antidepressant Advisor (ADeSS): A Decision Support System for Antidepressant Treatment for Depression in UK primary care - a feasibility study

Journal:	*BMJ Open*
Manuscript ID	bmjopen-2019-035905.R1
Article Type:	Protocol
Date Submitted by the Author:	04-Mar-2020

Complete List of Authors:	Harrison, Phillippa; Centre for Affective Disorders, Department of Psychological Medicine, Institute of Psychiatry, Psychology & Neuroscience, King's College London<br>Carr, Ewan ; Department of Biostatistics and Health Informatics, Institute of Psychiatry, Psychology & Neuroscience, King's College London<br>Goldsmith, Kimberley; Department of Biostatistics and Health Informatics, Institute of Psychiatry, Psychology & Neuroscience, King's College London<br>Young, Allan; Centre for Affective Disorders, Department of Psychological Medicine, Institute of Psychiatry, Psychology & Neuroscience, King's College London<br>Ashworth, Mark; Department of Population Health Sciences, King's College London<br>Fennema, Diede; Centre for Affective Disorders, Department of Psychological Medicine, Institute of Psychiatry, Psychology & Neuroscience, King's College London<br>Barrett, Barbara; Department of Health Services & Population Research, Institute of Psychiatry, Psychology & Neuroscience, King's College London<br>Zahn, Roland; Centre for Affective Disorders, Department of Psychological Medicine, Institute of Psychiatry, Psychology & Neuroscience, King's College London

**Primary Subject Heading**:	Mental health
Secondary Subject Heading:	Evidence based practice
Keywords:	Depression & mood disorders < PSYCHIATRY, PRIMARY CARE, Adult psychiatry < PSYCHIATRY

SCHOLARONE™
Manuscripts

I, the Submitting Author has the right to grant and does grant on behalf of all authors of the Work (as defined in the below author licence), an exclusive licence and/or a non-exclusive licence for contributions from authors who are: i) UK Crown employees; ii) where BMJ has agreed a CC-BY licence shall apply, and/or iii) in accordance with the terms applicable for US Federal Government officers or employees acting as part of their official duties; on a worldwide, perpetual, irrevocable, royalty-free basis to BMJ Publishing Group Ltd ("BMJ") its licensees and where the relevant Journal is co-owned by BMJ to the co-owners of the Journal, to publish the Work in this journal and any other BMJ products and to exploit all rights, as set out in our licence.

The Submitting Author accepts and understands that any supply made under these terms is made by BMJ to the Submitting Author unless you are acting as an employee on behalf of your employer or a postgraduate student of an affiliated institution which is paying any applicable article publishing charge ("APC") for Open Access articles. Where the Submitting Author wishes to make the Work available on an Open Access basis (and intends to pay the relevant APC), the terms of reuse of such Open Access shall be governed by a Creative Commons licence – details of these licences and which Creative Commons licence will apply to this Work are set out in our licence referred to above.

Other than as permitted in any relevant BMJ Author's Self Archiving Policies, I confirm this Work has not been accepted for publication elsewhere, is not being considered for publication elsewhere and does not duplicate material already published. I confirm all authors consent to publication of this Work and authorise the granting of this licence.

For peer review only

Study Protocol for The Antidepressant Advisor (ADeSS): A Decision Support System for Antidepressant Treatment for Depression in UK primary care - a feasibility study

Short Title: The Antidepressant Advisor

Phillippa Harrison[1], Ewan Carr[2], Kimberley Goldsmith[2], Allan H. Young[1], Mark Ashworth[3], Diede Fennema[1], Barbara Barrett[4], Roland Zahn[1]

[1]Centre for Affective Disorders
Department of Psychological Medicine
Institute of Psychiatry, Psychology & Neuroscience
King's College London
16 De Crespigny Park
London SE5 8AF

[2]Department of Biostatistics and Health Informatics
Institute of Psychiatry, Psychology & Neuroscience
King's College London
16 De Crespigny Park
London SE5 8AF

[3]Department of Population Health Sciences,
King's College London
Guy's Campus
London SE1 1UL

[4]Department of Health Services & Population Research,
Institute of Psychiatry, Psychology & Neuroscience
King's College London
David Goldberg Centre
De Crespigny Park
London SE5 8AF

Corresponding author:
Dr Roland Zahn
Centre for Affective Disorders
Department of Psychological Medicine
Institute of Psychiatry, Psychology & Neuroscience
King's College London
PO72, De Crespigny Park, London SE5 8AF
Telephone: +44 (0)20 7848 0348
E-mail: roland.zahn@kcl.ac.uk

Word count: 4133

For peer review only

Acknowledgements

This paper represents independent research funded by the National Institute for Health Research (NIHR) research for patient benefit scheme. This paper represents independent research part funded (KG, EC) by the National Institute for Health Research (NIHR) Biomedical Research Centre at South London and Maudsley NHS Foundation Trust and King's College London. The views expressed are those of the authors and not necessarily those of the NHS, the NIHR or the Department of Health and Social Care. DF's PhD is funded by the Medical Research Council Doctoral Training Partnership (project reference: 2064430). The authors would like to acknowledge the support provided to the study by the South London Clinical Research Network and sponsorship by Lambeth CCG. Additionally, the authors wish to thank the following members of the Trial Steering Committee who have dedicated their time and provided valuable input to the study: Dr Daniel Dietch, Dr Victoria Cornelius, Evelyn London and Dr Sarah Markham. We are also grateful to EMIS PLC with whom we have designed the software implementation of our Antidepressant Advisor decision support tool and to Alloc Modulo LTD with whom we have developed the accompanying MooDoC mobile app.

For peer review only

Abstract

Introduction: The Antidepressant Advisor Study is a feasibility trial of a computerised decision-support tool which uses an algorithm to provide antidepressant treatment guidance for General Practitioners (GPs) in the UK primary care service. The tool is the first in the UK to implement national guidelines on antidepressant treatment guidance into a computerised decision support tool.

Methods and analysis: The study is a parallel group, cluster-randomised controlled feasibility trial where participants are blind to treatment allocation. GPs were assigned to two treatment arms: 1) treatment as usual, 2) computerised decision support tool to assist with antidepressant choices. The study will assess recruitment and lost to follow-up rates, GP satisfaction with the tool, and impact on health service use. A meaningful long-term roll-out unit cost will be calculated for the tool and service use data will be collected at baseline and follow-up to inform a full economic evaluation of a future trial.

Ethics and dissemination: The study has received NHS ethical approval from the London - Camberwell St Giles Research Ethics Committee (ref:17/LO/2074). The trial was pre-registered in the Clinical Trials.gov registry. The results of the study will be published in a pre-publication archive within one year of completion of the last follow-up assessment.

Trial registration number: ClinicalTrials.gov Identifier: NCT03628027

Strengths and limitations of this study:

- A strength of the study is integration of a decision support tool into an existing UK primary care computer system to reduce the burden on GPs of adopting a new treatment system
- A further strength is that the study allows patients to be treated by their regular GP practice which reduces the burden on patients
- A limitation of the study is the potential for selection bias towards those with less severe depression who are well enough to attend study assessments

For peer review only - http://bmjopen.bmj.com/site/about/guidelines.xhtml

Introduction

Background & Rationale

Depression is a leading cause of disability world-wide [1] with approximately two million adults in the UK experiencing an episode each week [2]. Population-based studies show that only 50% of people with major depressive disorder (MDD) are treated [3; 4] and more than half of these receive inadequate treatment [4]. Furthermore, less than one third of patients fully recover after treatment with a standard antidepressant, such as citalopram [5], and only 40% of patients recover after accessing the National Health Service (NHS) Improving Access to Psychological Therapies (IAPT) programme [6]. The UK National Institute for Health and Care Excellence (NICE) recommends three first-line antidepressants with similar mechanism of action (fluoxetine, sertraline, and citalopram) [7]. Second- and third-line treatments are recommended by NICE, but, due to a lack of sequenced guidance, it is unclear whether and when GPs should prescribe them [7]. Overall, unlike for the management of other long-term conditions, there is no structured disease management for antidepressant prescribing in UK primary care [8; 9] and patients' access to second- and third-line antidepressants is variable. National prescription data show frequent and rising use of certain second-line antidepressants such as venlafaxine and mirtazapine without clear decision strategies [10]. A lack of structure in treatment decision-making and poor implementation of existing guidelines could exacerbate the suffering caused by insufficiently treated depression [4]. Enhanced depression care has been called for and entails tailoring treatment to measured outcomes [9].

One way to provide structured treatment guidelines is through algorithms which incorporate various patient characteristics and allocate treatments most likely to be effective. To the authors' knowledge, there is no scientifically evaluated and pragmatic antidepressant decision support tool in UK primary care. This is based on a literature search conducted in September 2019 in the ISRCTN, Eudra CT, Clinical Trials.gov, PubMed and Web of Knowledge databases using the key words: ("depression or depressive") AND "decision" AND ("computer or algorithm") AND "antidepressant". The search returned four relevant studies which employed a computer-based algorithm to help make decisions about antidepressant treatments [11-14].

Out of three completed studies, two found evidence for the effectiveness of algorithm support compared to treatment-as-usual. Adli et al. (2017) [13] found shorter time to remission and fewer medication switches using an algorithm compared to computerised support and treatment-as-usual for depression treatment. Kurian et al. (2009) [11] assigned private GPs and

For peer review only - http://bmjopen.bmj.com/site/about/guidelines.xhtml

their MDD patients to active computerised decision support for antidepressants based on the 1999 Texas Medication Algorithm Project (TMAP) [15] and treatment-as-usual. The active group showed a 41% response rate to treatment vs. 26% in the treatment-as-usual group. However, Rollman et al. (2002) [12] reported no difference in depressive symptoms after six months between active decision-support, non-specific prompting that depression needs treatment and treatment-as-usual. The ongoing Assurex Health Inc study [14] uses a genetic testing decision support tool to predict differential treatment response to treatments for depression.

Overall, all of the above studies were conducted outside of the UK, often in private healthcare settings which are difficult to compare with the UK NHS. Several of the support tools did not provide specific treatment sequences, only broad recommendations to change medication. Furthermore, some of the studies could not translate recommendations into algorithms to be used by GPs so that researchers had to manually provide GPs with advice reports. This creates a complicated treatment process and it seems unlikely that the support tools could provide ongoing recommendations to prescribers after initial treatment recommendations. Moreover, Kurian et al. (2009) [11] and Rollman et al. (2002) [12] employed algorithms that are now outdated and lacked integration of recent evidence on superiority of specific novel antidepressants in terms of tolerability and efficacy. All of the tools lacked integration into existing electronic healthcare record systems that GPs are familiar with. The NICE guidelines provide useful MDD treatment advice for GPs but a scarcity of studies on complex interventions using sequenced treatment algorithms has meant that the guidelines are unclear about how many switches between individual antidepressants are recommended before seeking specialist advice on other strategies i.e. augmentation. The seminal STAR*D study investigating such a sequenced treatment algorithm has used augmentation strategies that according to NICE should not be carried out by GPs [16].

Therefore, the aim of the current study is to assess the feasibility of a future definitive randomised controlled trial to test the efficacy and cost-effectiveness of a decision tool incorporating an algorithm to advise GPs on antidepressant prescribing for patients who have not responded to first-line treatments. In contrast to earlier work, the treatment algorithm is based on NICE guidelines including the latest health technology appraisals. If successful, the present study will therefore enable the first investigation into whether a sequenced treatment algorithm that relies on well-tolerated single antidepressants is effective and cost-effective. The

For peer review only - http://bmjopen.bmj.com/site/about/guidelines.xhtml

authors have gained support from one of the leading UK providers of primary care electronic record systems (EMIS group) who have implemented the algorithm into their software. The tool will be designed to be easy to use and to save time for GPs who are often limited to 10-minute appointments.

Study Objectives

1) To assess the feasibility of a future confirmatory trial investigating the first computerised decision support tool for antidepressant treatment in UK primary care by:
    a. estimating lost to follow-up rates;
    b. estimating GP adherence to the algorithm and patient adherence to prescribed medications;
    c. determining the number of GP practices willing to recruit patients for the study (determined by the Clinical Research Network (CRN) who will approach all practices in the participating Clinical Comissioning Groups (CCG));
    d. estimating participant recruitment rates per GP;
    e. estimating GP satisfaction with the decision tool.
2) To provide initial standard deviation estimates and intra-class correlation coefficients for computing effect size estimates for a larger confirmatory trial.
3) To collect health economic estimates of the roll-out cost of the intervention and changes in service use associated with the tool, including psychiatric referrals to mental health teams and/or the study psychiatrist.

Methods

Trial Design

The study design is a single-blinded cluster-randomised controlled clinical trial, where the GP practice is the cluster/randomisation unit. Considering complex intervention guidance [17], the study is designed as a feasibility study of the decision support tool to provide estimates of the unknown variables needed to plan a subsequent larger trial.

Study Setting

For peer review only

The study is based in UK primary care, recruiting GPs and patients from South London primary care practices. Patients will be assessed at the Institute of Psychiatry, Psychology and Neuroscience in South London and treated at their usual GP practice. The study start date was August 2018 and the planned end date is July 2021.

Participants

Eligibility criteria for patients

Inclusion criteria: age ≥ 18; at least moderately severe major depressive syndrome on Patient Health Questionnaire (PHQ-9; score≥15) [18]; no plans to change GP practice; able to complete self-report scales orally or in writing; no previous prescription of mirtazapine or vortioxetine; and early treatment resistance. The latter is defined as (i) current or recent prescription (in previous 2 months) of any of the following antidepressants: citalopram, fluoxetine, sertraline, escitalopram, paroxetine, venlafaxine, or duloxetine; and (ii) previous prescription of at least one other antidepressant from the same list.

Exclusion criteria: inability to consent to the study; unstable medical condition; currently being treated by mental health specialist; high suicide risk (Mini International Neuropsychiatric Interview (MINI suicidality screen)) [19]; past diagnosis of schizophrenia or schizo-affective disorder; current psychotic symptoms (three clinical screening questions to exclude schizophreniform disorders [20; 21]); bipolar disorder (WHO Composite International Diagnostic Interview (CIDI) screening scale for bipolar disorder [22]); currently at risk of being violent; drug or alcohol abuse over the last six months (Primary Care Evaluation of Mental Disorders (PRIME-MD [23]), modified to screen for drug abuse); breastfeeding/ within six months of giving birth; and both escitalopram and sertraline have already been prescribed.

Participant timeline

Potential participants will be identified using an EMIS eligibility tool to search participating GP practice medical records for patients meeting inclusion criteria (see Supplementary file 1). Interested participants will complete a pre-screening assessment, followed by a screening assessment including a clinical evaluation to determine full eligibility. The results of the screening assessment will be shared with GPs and patients. Inclusion decisions will not be made by GPs to help mitigate against selection biases and will be decided by the research team after the screening assessment. After being enrolled, patients receive access to a secure mobile phone

For peer review only - http://bmjopen.bmj.com/site/about/guidelines.xhtml

app developed in the study. Eligible patients will undergo treatment for depression over 14 weeks with their GPs. In both arms, patients will be seen by their GP who will review and monitor treatment. Patients' participation will last for approximately 15-18 weeks after their screening assessment, at which point their follow-up assessment will be conducted. See Table 1 below for participant timeline.

Table 1- Participant Timeline

	Participants identified by EMIS search tool	Consent for contact	Initial Pre-screening (electronic, letter, or phone)	Pre-Trial Screening Assessment in person	Treatment Sessions arranged by GP as soon as possible after pre-trial assessment with no fixed number over 14 weeks	Post-Trial assessment in person after 15-18 weeks since Pre-Trial assessment
Participants contacted about study	X					
Reply by letter slip, phone, text or email		X				
Oral/electronic or written pre-screening informed consent			X			
Introduction to the study			X			
Assessment of eligibility			X	X		
Written informed consent				X		
Clinical assessment & neuro-psychological tests				X		X
Trial intervention delivered by GPs					X	
Mobile app/ phone weekly assessment					X	

For peer review only - http://bmjopen.bmj.com/site/about/guidelines.xhtml

Interventions

Participants meeting the eligibility criteria will participate in the intervention arm to which their GP practice is randomised:

Arm 1: Treatment-as-usual (TAU). Treatment as GPs would usually deliver with no decision support and no constraints (these GPs will not be aware of the rules of the treatment algorithm used in the other group nor will they be able to access it);

Arm 2: Decision Support Tool. Treatment using the decision support tool to assist GPs with antidepressant prescriptions, prompting GPs to review patients' medications and change them if ineffective. The algorithm and technical requirements of the tool are described below.

An EMIS decision support module was developed which provides suggestions for antidepressant prescribing after highlighting the importance of non-pharmacological strategies such as psychological therapy or exercise. The treatment recommendations are based on NICE guidelines, British Association for Psychopharmacology guidelines [24] and Maudsley prescribing guidelines [25]. The algorithm is designed to incorporate patient preferences, ensure maximum prescribing safety and offer guidance on antidepressant monotherapy (avoiding combinations). The NICE guidelines state that GPs should not normally initiate combinations without consulting a psychiatrist first [7]. See Figure 1 for stepped recommendations provided in the tool; for further detail see Supplementary file 1.

(please insert Figure 1 here)

The module is implemented only for those GP practices taking part in the study and only for participating patients at those practices. GPs will use the EMIS module during patient appointments by entering patients' Maudsley Modified Patient Health Questionnaire (MM-PHQ-9) [26] scores of their depressive symptoms over the last week from the study mobile app. The tool will offer treatment advice on whether a patient's antidepressant should be maintained or changed and what it should be changed to. The advisor tool is used for every depression review appointment over the 14-week study duration, with no minimum number of appointments.

GPs in both conditions will receive training by the study Principal Investigator (PI) on study procedures including adverse event reporting and incorporating patients' mobile app data. Training will be tailored to treatment arm so that GPs in the decision support tool arm will be

For peer review only - http://bmjopen.bmj.com/site/about/guidelines.xhtml

trained in how to use the EMIS module to guide treatment decisions whereas GPs in the TAU arm will be advised to use their clinical judgement as usual for treatment.

Adherence

Adherence to the prescribed drugs in both arms will be monitored using the available tools on EMIS as well as the mobile app. Patients will be withdrawn from the antidepressant early in the event of a serious or medically important adverse event considered by the GP to be related to the intervention, hypomania, or a significant deterioration of symptoms requiring referral to secondary care. GP adherence to the algorithm will be measured after GPs have completed their participation in the study with a self-report questionnaire and information from EMIS.

Outcomes

Feasibility outcome measures:

1) Lost to follow-up rates;
2) GP adherence to the algorithm for each completed patient rated by a trial clinician (0 – 3 for none, less than 50%, 50% or more or 100% of recommended steps implemented);
3) Average patient adherence to prescribed medications based on EMIS electronic prescribing records;
4) Adverse Event (AE) and Serious Adverse Event (SAE) rates (grade and relationship to intervention);
5) Patient adherence to GP attendance measured by % of attended GP visits out of scheduled visits on EMIS over treatment period;
6) Recruitment rates;
7) Average GP satisfaction with decision support tool (intervention arm; after GP completion of study);
8) DSM-IV Social and Occupational Functioning Assessment Scale (SOFAS) scale of psychosocial functioning (on final visit, whilst modelling baseline score) [27];
9) Maudsley Visual Analogue Mood Scale (on final visit, whilst modelling baseline score);

This section describes outcomes that will be measured in this feasibility trial to provide estimates (e.g. effect size, standard deviation) needed for sample size calculations for a future

For peer review only - http://bmjopen.bmj.com/site/about/guidelines.xhtml

confirmatory trial. These outcomes will not be used for inferential comparisons between trial arms.

Primary clinical outcomes for a future confirmatory trial:

10) Self-rated Quick Inventory of Depressive Symptomatology sum score (QIDS-SR16) [28] at final visit, adjusting for baseline score;

Secondary clinical outcome measures for a future confirmatory trial:

11) Depressive symptoms assessed by the Montgomery–Asberg Depression Rating Scale (MADRS) at follow-up assessment, adjusting for baseline score [29];
12) Clinical Global Impression scale; change between baseline and follow-up assessment (final visit) [30];
13) Generalised Anxiety Disorder (GAD-7) scale at follow-up assessment, adjusting for baseline score [23];
14) Body Mass Index at follow-up assessment adjusting for baseline score;

Exploratory clinical outcome measures (self-report via mobile app)

15) Average score for medication side effects on Frequency, Intensity and Burden of Side Effects Ratings (FIBSER) [31];
16) Average % of adherence to prescribed antidepressant medication;
17) Average MM-PHQ-9 scores in last 2 weeks (at follow-up, whilst modelling first 2 weeks as baseline average) [26];

Health Economic measures:

18) Service Use as determined on EMIS including psychiatric referrals and referrals to study psychiatrist, as well as time to psychiatric referral; also primary care consultation rates;
19) Service Use; self-reported using a modified version of the Adult Service Use Schedule (AD-SUS) [32];
20) Quality of life using the EQ-5D-3L [33] - the standard measure recommended by NICE for cost-effectiveness analyses.

Sample size

Each practice will be asked to enrol approximately 8-11 participants. The study aims to enrol 86 participants assuming the same lost to follow-up rate as in Kurian et al.'s (2009) study

For peer review only - http://bmjopen.bmj.com/site/about/guidelines.xhtml

[11](18%), giving a final sample size of 70 (35 in each group as recommended [34]). This will enable estimation of the lost to follow-up rate within a 95% confidence interval of ± 8% [35]. Kurian et al. [11] did not provide effect size estimates so the present study has been designed to provide means and standard deviations, as well as confidence intervals for measures of change on the primary outcome measure (QIDS-SR16 [28]) as recommended for feasibility trials [34].

Recruitment of GP Practices

The study aims to recruit 8-20 GP practices. Only one GP per practice will be recruited to the study to avoid communication about treatment allocation between GPs. In the event of a GP leaving a participating practice, an alternative GP from the same practice will replace them.

Enrolment of Participants

Patients will be enrolled after GPs are randomised. An EMIS eligibility tool developed for the study will identify potential participants from each practice meeting the inclusion/exclusion criteria (see Supplementary file 1 for further details). Practice/CRN staff will run the EMIS search and send eligible patients a study advert and request for consent for contact via post.

Assignment of Interventions

Allocation

Sequence Generation: The randomisation service will be provided by the King's Clinical Trials Unit (KCTU) in accordance with a standard operating procedure and held on a secure server. GP practices will be randomised in pairs with a 1:1 allocation to either intervention or TAU using block randomisation with random permuted blocks of block size two. This will help maintain allocation concealment while ensuring a similar number of practices are allocated to each arm.

Concealment Mechanism: KCTU will send the randomisation outcome to unblinded researchers only to ensure researchers conducting assessments remain blind. The randomisation details will be kept on a password-protected network drive only accessible by the PI.

Blinding

Patients, researchers assessing observer-rated outcomes and the senior statistician will be blind to treatment allocation. All other members of the study team, including the junior statistician,

For peer review only - http://bmjopen.bmj.com/site/about/guidelines.xhtml

and GPs will be unblinded. Unblinding incidents will be recorded in the trial master file and reported to the Trial Steering Committee (TSC; see Data Monitoring section). Unblinded assessments will be retained in the study. If a researcher becomes unblinded prior to completing assessments, an alternative researcher will conduct future assessments with patients from that practice in order to conduct unbiased assessments. There is no requirement for an emergency unblinding procedure, as the PI and GPs are not blinded.

Data Collection Methods

The pre-screening assessment is conducted online using a survey software (alternatively over the telephone or via postal survey) and includes the PRIME-MD (self-report) [23] to assess depressive symptoms and alcohol abuse and modified to screen for drug abuse, the CIDI (self-report) for bipolar disorder [22], questions to exclude schizophreniform disorders and psychotic depression [20; 21], and pregnancy in women. Eligible participants are invited to a face-to-face screening assessment which includes depression history, treatment history, medical history, MINI suicidality screen [19], DSM-5 Structured Clinical Diagnostic Interview [27], Psychiatric Family History Screen [36], AMDP Psychopathology Interview questions of depression items [37; 38], Addenbrooke's Cognitive Examination-III in patients over 50 to detect early Alzheimer's disease [39], Longitudinal Interval Follow-up Interview (LIFE) [40], and the Young Mania Rating Scale (YMRS) [41]. Participants' medical records will be checked to confirm medical details. Participants will use the mobile app for the 14-week study duration to enter weekly MM-PHQ-9 ratings [26], hypomanic symptoms [22], FIBSER medication side effects [31], medication changes and self-blame-related action tendencies such as to what extent participants would feel like hiding or creating a distance from themselves (using two questions developed in Dr Zahn's research lab). A daily question will ask about medication adherence. Alternatively, participants can opt to provide this data at weekly intervals via post or phone.

At the follow-up assessment, the study outcome measures and YMRS [41] will be repeated. The LIFE [40] will be used to determine remission and its psychiatric status rating scale used as a comparison to baseline. All assessments will be conducted by trained researchers. The research team will be trained and closely supervised, establishing sufficient inter-rater reliability on semi-structured interviews with the PI before conducting assessments independently [21].

Patient and Public Involvement

For peer review only - http://bmjopen.bmj.com/site/about/guidelines.xhtml

The study is supported by a service user advisory group which provides input to the study. This group meets on a regular basis and provides insight into the study design, information disseminated to patients and the burden of trial participation from the patient's perspective. At the end of the study, the service user advisory group will comment on the findings and contribute to the dissemination plan.

Statistical Methods

Outcomes

Categorical outcomes (e.g. loss to follow-up) will be described using frequencies and proportions. The QIDS-SR16 and other continuous outcomes will be summarised at baseline and follow-up using means and standard deviations. The GP practice intra-class correlation will be calculated for the outcome variable using one-way random effects analysis of covariance (adjusted for baseline). A preliminary analysis of the difference between the groups, as far as possible using the intention-to-treat principle, is planned. This analysis will be identified as preliminary and underpowered when published, and no *p* values will be provided. Continuous outcomes measured at baseline and follow-up only, such as the QIDS-SR16, will be analysed using linear regression with robust standard error calculation to account for clustering within GP practice. Continuous outcomes measured weekly will be analysed using mixed linear regression models with a random intercept for GP practice to account for clustering. Both sets of models will include treatment arm as a covariate and will adjust for baseline measure of the outcomes where appropriate. Any missing baseline data will be imputed using mean imputation [42]. Missing data in weekly outcomes will be accounted for under the missing at random assumption by using the maximum likelihood algorithm to fit the mixed models. We may consider multiple imputation for outcomes measured only at follow-up if post-randomisation variables can be quantified and are related to having missing outcomes. Adverse events (see Harms section) will be summarised separately as AEs and SAEs, by intervention group, as number of events and number of people experiencing events.

Data Monitoring

The TSC will meet bi-annually to oversee study progress and comprises the study PI, research associate, research assistants, statisticians, collaborators and service users. There are no formal criteria for terminating the trial. Informal decisions about early trial termination will be made by the TSC and sponsor should they have major concerns about safety or conduct of the trial.

For peer review only

For peer review only - http://bmjopen.bmj.com/site/about/guidelines.xhtml

This would be considered in the event that extensive recurrent serious or medically important adverse events are observed, particularly if these are Suspected Unexpected Serious Adverse Reactions (SUSARs).

Harms

GPs participating in the study, especially in the intervention arm, are likely to change medications more often and prescribe medications which are less frequently used in primary care. All medications recommended by the decision support tool are in accordance with NICE guidance and were carefully selected for the study based on likelihood of positive effects and side effects (see Supplementary file 1). Kurian et al. (2009) [11] found their support tool to be beneficial for depression treatment, although some patients felt worse as a result of changing medications – for example, having more suicidal thoughts. Such side effects will be monitored by GPs and discussed with patients.

AEs/SAEs are defined in Supplementary file 1. AEs/SAEs will be recorded from the time the subject provides informed consent at the baseline assessment until their follow-up. GPs will record all AEs/SAEs on EMIS and they will be regularly collected from GPs by the research team and assessed. AEs/SAEs reported to the PI will be recorded on AE forms as part of the case report form using an ID number to identify the patient. AEs/SAEs will be reported bi-annually to the TSC and an unblinded sub-committee, assuming the functions of the data monitoring committee, will review the information separated by arm.

Ethics and Dissemination

Research Ethics Approval

The study has received NHS ethical approval from the London - Camberwell St Giles Research Ethics Committee (ref:17/LO/2074).

Protocol Amendments

Modifications to the protocol will be conveyed to the TSC at the planning stage for members to provide input, then again to inform the committee of the ethics committee decision.

Consent

Participants will provide informed consent to take part in the study and are free to withdraw at any time with no reason given (see Supplementary files 1 & 2 for further detail).

For peer review only

Dissemination

The trial has been pre-registered on ClinicalTrials.gov (Identifier: NCT03628027). See Supplementary file 1 for further detail on dissemination.

Health economics

The study will not include a formal economic evaluation, instead the focus is on developing the tools needed to support a future trial. First, a meaningful unit cost will be calculated for our tool in collaboration with EMIS. The unit cost will consider the long-term roll-out cost of the intervention and investigate how to allocate this to an individual patient in order to carry out a future cost-effectiveness analysis. Second, the methods for the collection of service use data will be optimised, which will include a comparison of data collected from EMIS and data collected from patients via self-report.

Limitations

One limitation of the algorithm is that it does not take pharmacogenomic evidence into account which could be an important refinement in the future, should pharmacogenomic testing become implemented into NHS routine care. For example, both CYP2D6 and CYP2C19 gene polymorphisms affect the liver metabolism of sertraline, escitalopram and citalopram, as well as paroxetine and fluvoxamine [43]. Therefore one could argue if someone has not responded to or not tolerated citalopram or paroxetine, this carries a higher risk of non-tolerance or non-response of escitalopram and sertraline. From a pragmatic perspective, however, it is worth trying escitalopram or sertraline as the first step in the algorithm, because pharmacogenomic effects of liver enzymes are only one of many factors which determine response and tolerance [44]. CYP2D6 also plays a role in metabolising vortioxetine and mirtazapine, but pharmacogenomic guidelines have not been developed for these to our knowledge.

Contributorship Statement

For peer review only - http://bmjopen.bmj.com/site/about/guidelines.xhtml

PH wrote the manuscript and EC, KG, AHY, MA, DF, BB and RZ all commented significantly on drafts of the manuscript. EC and KG provided the statistical analysis plan, BB provided the health economic analysis plan and AHY, MA and RZ provided oversight on the study procedures and delivery.

Competing Interests

AHY is employed by King's College London as an honorary consultant in the South London and Maudsley Trust (NHS UK) and is a consultant to Johnson & Johnson and Livanova. AHY has given paid lectures and sat on advisory boards for the following companies with drugs used in affective and related disorders: Astrazenaca, Eli Lilly, Lundbeck, Sunovion, Servier, Livanova, Janssen, Allegan, Bionomics, Sumitomo Dainippon Pharma. AHY has received honoraria for attending advisory boards and presenting talks at meetings organised by LivaNova. AHY is the Principal Investigator of the following studies: Restore-Life VNS registry study funded by LivaNova, ESKETINTRD3004: "An Open-label, Long-term, Safety and Efficacy Study of Intranasal Esketamine in Treatment-resistant Depression," "The Effects of Psilocybin on Cognitive Function in Healthy Participants," and "The Safety and Efficacy of Psilocybin in Participants with Treatment-Resistant Depression (P-TRD)." AHY has received grant funding (past and present) from the following: NIMH (USA); CIHR (Canada); NARSAD (USA); Stanley Medical Research Institute (USA); MRC (UK); Wellcome Trust (UK); Royal College of Physicians (Edin); BMA (UK); UBC-VGH Foundation (Canada); WEDC (Canada); CCS Depression Research Fund (Canada); MSFHR (Canada); NIHR (UK); Janssen (UK). AHY has no shareholdings in pharmaceutical companies.

RZ is a private psychiatrist service provider at The London Depression Institute and co-investigator on a Livanova-funded observational study of Vagus Nerve Stimulation for Depression. RZ has received honorarium for talks at three medical symposia sponsored by

For peer review only

Lundbeck as well as a talk funded by Janssen whom he collaborates with on an MRC-funded grant. KG reports grants from NIHR, Stroke association, National Institutes of Health (US) and Juvenile Diabetes Research Foundation (US) during the conduct of the study. EC reports personal fees from NIHR during the conduct of the study. BB reports grants from NIHR Research for Patient Benefit during the conduct of the study. MA, PH and DF report no competing interests.

For peer review only

Funding

The research has received funding from the National Institute for Health Research (NIHR) research for patient benefit (RfPB) (grant reference: PB-PG-0416-20039), the National Institute for Health Research (NIHR) Biomedical Research Centre at South London and Maudsley NHS Foundation Trust and King's College London and the Medical Research Council Doctoral Training Partnership (grant reference: 2064430).

Data Sharing Statement

The trial is ongoing so data sharing is not currently available.

References

1. WHO. The global burden of disease: 2004 Update. 2008.
2. UK National Centre for Social Research. Adult Psychiatric Morbidity Survey. 2007.
3. Dormon F. Is mental health care improving?: The Health Foundation. 2015.
4. Kessler RC, Berglund P, Demler O et al. . The epidemiology of major depressive disorder: results from the National Comorbidity Survey Replication (NCS-R). JAMA 2003;289(23):3095-105.
5. Trivedi MH, Rush AJ, Wisniewski SR et al. . Evaluation of outcomes with citalopram for depression using measurement-based care in STAR*D: implications for clinical practice. Am J Psychiatry 2006;163(1):28-40.
6. Gyani A, Shafran R, Layard R, Clark DM. Enhancing recovery rates: lessons from year one of IAPT. Behav Res Ther 2013;51(9):597-606.
7. NICE. Depression: The treatment and management of depression in adults; 2010. City: The British Psychological Society and The Royal College of Psychiatrists.
8. Symons L, Tylee A, Mann A et al. . Improving access to depression care: descriptive report of a multidisciplinary primary care pilot service. Br J Gen Pract 2004;54(506):679-83.
9. Von Korff M, Goldberg D. Improving outcomes in depression. BMJ 2001;323(7319):948-9.

For peer review only - http://bmjopen.bmj.com/site/about/guidelines.xhtml

10. Spence R, Roberts A, Ariti C, Bardsley M. Focus On: Antidepressant prescribing. The Health Foundation & Nuffield Trust; 2014.
11. Kurian BT, Trivedi MH, Grannemann BD et al. . A computerized decision support system for depression in primary care. Prim Care Companion J Clin Psychiatry 2009;11(4):140-6.
12. Rollman BL, Hanusa BH, Lowe HJ et al. . A randomized trial using computerized decision support to improve treatment of major depression in primary care. J Gen Intern Med 2002;17(7):493-503.
13. Adli M, Wiethoff K, Baghai TC et al. . How effective is algorithm-guided treatment for depressed inpatients? Results from the randomized controlled multicenter German Algorithm Project 3 trial. International Journal of Neuropsychopharmacology 2017;20(9):721-730.
14. Inc AH. Pharmacogenomic Decision Support With GeneSight Psychotropic to Guide the Treatment of Major Depressive Disorder. ClinicalTrials.gov registered 2015.
15. Crismon ML, Trivedi M, Pigott TA et al. . The Texas Medication Algorithm Project: report of the Texas Consensus Conference Panel on Medication Treatment of Major Depressive Disorder. J Clin Psychiatry 1999;60(3):142-56.
16. Rush AJ, Fava M, Wisniewski SR et al. . Sequenced treatment alternatives to relieve depression (STAR* D): rationale and design. Controlled clinical trials 2004;25(1):119-142.
17. MRC. Developing and evaluating complex interventions: new guidance. 2006.
18. Spitzer RL, Kroenke K, Williams JW, and the Patient Health Questionnaire Primary Care Study G. Validation and utility of a self-report version of prime-md: The phq primary care study. JAMA 1999;282(18):1737-1744.
19. Sheehan DV, Lecrubier Y, Sheehan KH et al. . The Mini-International Neuropsychiatric Interview (M.I.N.I.): the development and validation of a structured diagnostic psychiatric interview for DSM-IV and ICD-10. J Clin Psychiatry 1998;59 Suppl 20:22-33;quiz 34-57.
20. Green MF, Hellemann G, Horan WP et al. . From perception to functional outcome in schizophrenia: modeling the role of ability and motivation. Archives of General Psychiatry 2012;69(12):1216-1224.
21. Lythe KE, Moll J, Gethin JA et al. . Self-blame–selective hyperconnectivity between anterior temporal and subgenual cortices and prediction of recurrent depressive episodes. JAMA psychiatry 2015;72(11):1119-1126.
22. Kessler RC, Akiskal HS, Angst J et al. . Validity of the Assessment of Bipolar Spectrum Disorders in the WHO CIDI 3.0. Journal of affective disorders 2006;96(3):259-269.
23. Spitzer RL, Williams JW, Kroenke K, et al. Utility of a new procedure for diagnosing mental disorders in primary care: The prime-md 1000 study. JAMA 1994;272(22):1749-1756.
24. Cleare A, Pariante CM, Young AH et al. . Evidence-based guidelines for treating depressive disorders with antidepressants: A revision of the 2008 British Association for Psychopharmacology guidelines. J Psychopharmacol 2015;29(5):459-525.
25. Taylor D, Paton C, Kapur S, South London and Maudsley NHS Trust. The Maudsley prescribing guidelines in psychiatry: Wiley. 2015.
26. Walton S. Developing a novel outcome measure for depression: The Maudsley Modified Patient Health Questionnaire-9. . Unpublished Masters dissertation, King's College London. 2019.
27. First MB SR, Gibbon M, Williams JBW. Structured Clinical Interview for DSM-IV-TR Axis I Disorders, Research Version, Patient Edition. (SCID-I/P). . New York, NY: Biometrics Research, New York State Psychiatric Institute 2002.
28. Rush AJ, Trivedi MH, Ibrahim HM et al. . The 16-Item Quick Inventory of Depressive Symptomatology (QIDS), clinician rating (QIDS-C), and self-report (QIDS-SR): a psychometric evaluation in patients with chronic major depression. Biol Psychiatry 2003;54(5):573-83.
29. Montgomery SA, Åsberg M. A new depression scale designed to be sensitive to change. British Journal of Psychiatry 1979;134(4):382-389.

For peer review only - http://bmjopen.bmj.com/site/about/guidelines.xhtml

30. Busner J, Targum SD, Miller DS. The Clinical Global Impressions scale: errors in understanding and use. Compr Psychiatry 2009;50(3):257-62.
31. Wisniewski SR, Rush AJ, Balasubramani GK et al. . Self-rated global measure of the frequency, intensity, and burden of side effects. J Psychiatr Pract 2006;12(2):71-9.
32. Kuyken W HR, Barrett B, BynG R, Dalgleish T, Kessler D et al. Effectiveness and cost-effectiveness of mindfulness-based cognitive therapy compared with maintenance antidepressant treatment in the prevention of depressive relapse or recurrence (PREVENT): a randomised controlled trial. . The Lancet. 2015;386:63-73.
33. R. B. EuroQol: the current state of play. Health Policy. 1996.;37:53-72.
34. Teare MD, Dimairo M, Shephard N et al. . Sample size requirements to estimate key design parameters from external pilot randomised controlled trials: a simulation study. Trials 2014;15:264.
35. Lwanga SK, Lemeshow S, Organization WH. Sample size determination in health studies: a practical manual. 1991.
36. Weissman MM WP, Adams P, Wolk S, Verdeli H, Olfson M. . Brief screening for family psychiatric history: the family history screen. . Arch Gen Psychiatry 2000;57:675.
37. Fähndrich E RE. The AMDP system for the documentation of psychiatric symptoms: course and effectivity of a training seminar. Pharmacopsychiatry 1985;18:278-281.
38. Fähndrich E SR. Leitfaden zur Erfassung des psychopathologischen Befundes: halbstrukturiertes Interview anhand des AMDP-Systems. Hogrefe Verlag 1989.
39. Noone P. Addenbrooke's cognitive examination-III. Occupational Medicine 2015;65(5):418-420.
40. Keller MB, Lavori PW, Friedman B et al. . The Longitudinal Interval Follow-up Evaluation. A comprehensive method for assessing outcome in prospective longitudinal studies. Arch Gen Psychiatry 1987;44(6):540-8.
41. Young RC, Biggs JT, Ziegler VE, Meyer DA. A rating scale for mania: reliability, validity and sensitivity. Br J Psychiatry 1978;133:429-35.
42. White IR, Thompson SG. Adjusting for partially missing baseline measurements in randomized trials. Stat Med 2005;24(7):993-1007.
43. Hicks JK, Bishop JR, Sangkuhl K et al. . Clinical Pharmacogenetics Implementation Consortium (CPIC) guideline for CYP2D6 and CYP2C19 genotypes and dosing of selective serotonin reuptake inhibitors. Clinical Pharmacology & Therapeutics 2015;98(2):127-134.
44. Bousman CA, Forbes M, Jayaram M et al. . Antidepressant prescribing in the precision medicine era: a prescriber's primer on pharmacogenetic tools. BMC psychiatry 2017;17(1):60.

For peer review only

Figure 1-Decision chart for decision support tool

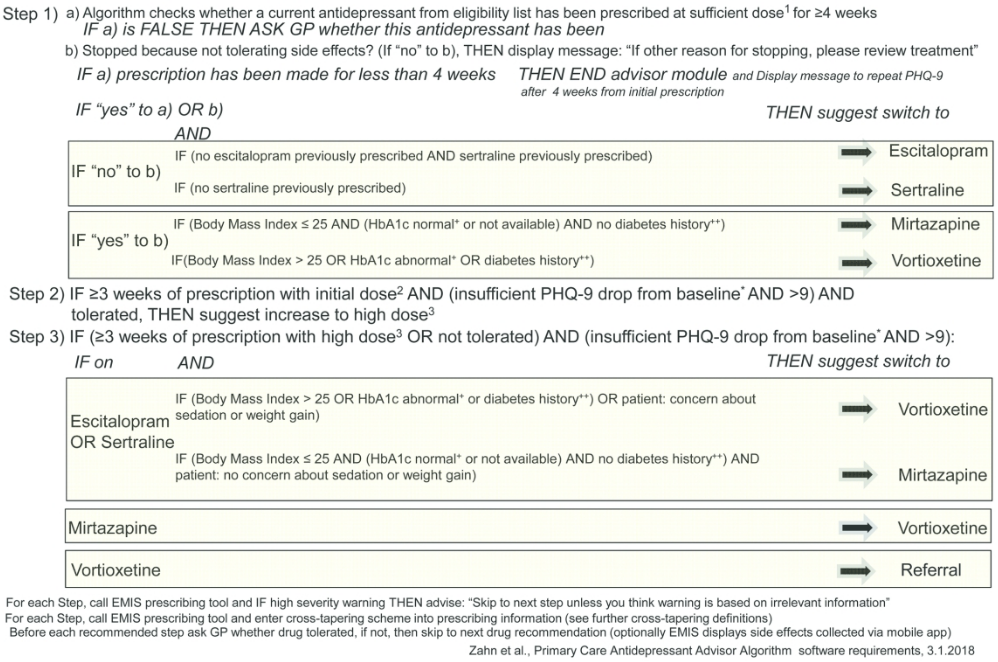

Figure 1-Decision chart for decision support tool

196x130mm (300 x 300 DPI)

SUPPLEMENTARY FILE 1

Study Protocol for The Antidepressant Advisor (ADeSS): A Decision Support System for Antidepressant Treatment for Depression in UK primary care - a feasibility study

Short Title: The Antidepressant Advisor

Supplementary Methods

EMIS Eligibility Tool Criteria

1) current or recent prescription (in the last 2 months) of any of the following antidepressants listed: citalopram, fluoxetine, sertraline, escitalopram, paroxetine, venlafaxine, or duloxetine
2) previous prescription of at least one other antidepressant out of the same list of antidepressants
3) exclude patients for whom both escitalopram and sertraline have already been prescribed
4) no previous prescription of mirtazapine or vortioxetine
5) no mania/hypomania/bipolar (or synonyms), psychosis/schizophrenia (or synonyms), or drug abuse/misuse/dependence (or synonyms), or alcohol abuse/misuse/dependence or synonyms
6) age≥18 years
7) not pregnant
8) not given birth in last 6 months.

Decision Support Tool Rationale & Details

The decision chart for the decision support tool is presented in Figure 1 below. A network meta-analysis, not including vortioxetine, showed that sertraline and escitalopram are superior in terms of acceptability and efficacy compared to other antidepressants [1]. Since sertraline is among the first–line options recommended by NICE, if patients tolerate serotonin reuptake inhibitors (SSRI), the algorithm starts with recommending escitalopram for those patients where two NICE recommended first-line treatments have failed including sertraline, but starts with sertraline should it not have been prescribed before. For patients who have not tolerated SSRIs (verified by GP), the algorithm makes alternative suggestions. Mirtazapine is among the most efficacious antidepressants [1] and has a different side effect profile to SSRIs which is why it is suggested for these patients. Because of its 75% risk in inducing weight gain and thereby increasing the risk of metabolic syndrome[2], the algorithm suggests

For peer review only - http://bmjopen.bmj.com/site/about/guidelines.xhtml

vortioxetine in those patients who are at an increased risk of metabolic syndrome (Body Mass Index(BMI)>25, HbA1c increased), because of its low weight-gain-induction risk[3]. Vortioxetine has SSRI properties, but is less likely to induce typical side effects, apart from nausea [3]. A recent NICE health technology appraisal [4]has given a positive recommendation for using vortioxetine in patients whose depressive episode has responded inadequately to two antidepressants. The REVIVE trial [3] showed the benefits of vortioxetine in patients with current MDD who had not responded to SSRI or dual-reuptake inhibitor monotherapy. The appraisal also concluded that vortioxetine may have a better overall safety profile than other antidepressants. For those patients tolerating SSRIs who do not respond to escitalopram and are at an increased metabolic syndrome risk, the algorithm therefore suggests vortioxetine.

Venlafaxine, which is among the most efficacious medications[1], was omitted from the algorithm, because of its higher risk of death in overdose which limits its use in primary care according to the Medicines and Healthcare Regulatory Agency [5]guidance and most GPs we discussed this with are not prepared to prescribe it at the higher doses at which it acts as a noradrenaline reuptake inhibitor. Duloxetine was excluded as an alternative as it was less efficacious in a network meta-analysis [1].

For escitalopram non-responders who are at low metabolic syndrome risk, the algorithm suggests mirtazapine as a next step. For mirtazapine non-responders, a switch to vortioxetine is suggested as a next step. For vortioxetine non-responders during the second or third step, a psychiatric referral to the study psychiatrist is suggested, who could then recommend higher side effect burdened alternatives such as high-dose venlafaxine or augmentation strategies according to NICE guidelines. Agomelatine has been omitted from the algorithm despite its suitability for patients who are not tolerating SSRIs, because there is no positive HTA recommendation for it and because it requires liver enzyme checks [6].

Data Management

Data will be entered electronically into a password-protected database and stored separately from identifiable information. Data validation will be applied to ensure that entered data follows the correct format and is within a valid range. Data will be stored electronically in the master folder and will only be accessed by members of the research team. Data will be

For peer review only - http://bmjopen.bmj.com/site/about/guidelines.xhtml

backed up by the research associate every time the database is added to on an external hard drive.

Harms

An adverse event (AE) is any untoward medical occurrence in a patient or healthy volunteer, which does not necessarily have a causal relationship with the study procedures. A Serious Adverse Event (SAE) is any undesirable medical occurrence in a participant that is life threatening, requires hospitalisation, results in persistent/significant disability/incapacity, causes a congenital anomaly/ birth defect or results in death.

These events may be spontaneously revealed by the subject or observed by the GP and will be recorded regardless of whether the GP believes them to be related to the intervention. The following aspects of AEs/SAEs will be assessed: symptom, possible diagnosis or syndrome, date and duration of AE/SAE, causal relationship with investigation (definite / probable / possible / unlikely / not related), outcome (resolved / resolving / not resolved / resolved with sequelae / fatal / unknown), treatment of event, if applicable (e.g. any medication as well as any other medical measures), actions taken regarding assessment.

GPs will be asked to report SAEs within 24 hours to Lambeth CCG and the PI via email or phone without details which are then followed up with a more detailed report. SAEs which according to the GP are clearly unrelated to the study intervention (e.g. hospitalization for an unrelated illness) and AEs expected when treating depression[i], even if there is a possible relation to the intervention, are exempt from this immediate reporting and will instead be reported together with other AEs at the earliest opportunity (at least quarterly). An executive committee formed of two members of the TSC (one independent) will discuss any AEs/SAEs prior to each TSC then report any concerns back to the TSC. Blinded members of the TSC will be asked to leave the room for the discussion of AEs/SAEs in order to remain blinded. Medically important events or SAEs will be assessed by a medically qualified doctor in the study team and discussed with the TSC. If possible, blinded doctors will assess serious or medically important events, whereas other AEs may be classified by a non-blinded doctor. Classification into SUSAR vs. expected ones will be based on the most updated versions of the summary product characteristics of the prescribed drugs on https://www.medicines.org.uk/emc/. SUSARs would be reported to the sponsor and ethics

For peer review only - http://bmjopen.bmj.com/site/about/guidelines.xhtml

committee. GPs will be asked to document all other AEs and reasons for withdrawing from the study on EMIS.

Consent

Consent and screening will be carried out in two stages. First, patients identified by the EMIS eligibility tool will be asked to provide written consent to participate in the online pre-screening assessment, with phone and postal options also offered. Second, patients found to be eligible at pre-screening will be invited to the baseline assessment where they will be asked to provide written consent to participate in the feasibility trial. Each participant will receive an oral and written explanation of the purposes, procedures, and risks of this study. Written consent will be obtained after explaining the goals, procedures, risks (and discomforts), benefits, and nature of the study to patients. Only adult volunteers and patients who are capable of understanding the study procedures will be allowed to sign the consent forms.

Participants can withdraw from the trial at any time and this will be made clear in the information sheets and consent forms. The reason for withdrawal does not need to be provided.

Dissemination Policy

Reporting bias will be avoided by a priori registration in a public trial register in addition to publication of the current trial protocol as the research is ongoing. All members of the TSC will have the opportunity to review and have input into the current protocol. The results of the study will be published in a pre-publication archive within one year after completion.

For peer review only - http://bmjopen.bmj.com/site/about/guidelines.xhtml

References

1. Cipriani A, Furukawa TA, Salanti G et al. Comparative efficacy and acceptability of 12 new-generation antidepressants: a multiple-treatments meta-analysis. Lancet 2009;373(9665):746-58.
2. Masand PS, Gupta S. Long-term side effects of newer-generation antidepressants: SSRIS, venlafaxine, nefazodone, bupropion, and mirtazapine. Ann Clin Psychiatry 2002;14(3):175-82.
3. Montgomery SA, Nielsen RZ, Poulsen LH, Häggström L. A randomised, double-blind study in adults with major depressive disorder with an inadequate response to a single course of selective serotonin reuptake inhibitor or serotonin–noradrenaline reuptake inhibitor treatment switched to vortioxetine or agomelatine. Human Psychopharmacology: Clinical and Experimental 2014;29(5):470-482.
4. NICE. Vortioxetine for treating major depressive episodes - Technology appraisal guidance. 2015.
5. (MHRA). MaHRA. Selective serotonin reuptake inhibitors (SSRIs) and serotonin and noradrenaline reuptake inhibitors (SNRIs): use and safety. . 2014.
6. Agomelatine (Valdoxan): risk of liver toxicity. . Medicines and Healthcare Regulatory Agency (MHRA). 2014.

[i] These include non-fatal suicide attempt, non-suicidal self-harm-related injury, non-fatal traffic accident under medication influence, treatment-emergent [hypo-]mania with associated non-fatal complications, non-fatal somatic or anxiety-related complaints leading to A&E visits).

For peer review only - http://bmjopen.bmj.com/site/about/guidelines.xhtml

SUPPLEMENTARY FILE 2

Participant Identification Number for this trial: **ADeSS-S1-**

Date of Birth:

CONSENT FORM

Study Title: Antidepressant Advisor – Study 1
Name of Chief Investigator: Dr Roland Zahn

Please initial:

1. I confirm that I have read and understand the information sheet dated 24 Jan 2018 (version 2) for the above study. I have had the opportunity to consider the information, ask questions and have had these answered satisfactorily.	☐
2. I understand that my participation is voluntary and that I am free to withdraw at any time without giving any reason, without my medical care or legal rights being affected.	☐
3. I understand that relevant sections of my medical notes and data collected during the study may be looked at by individuals from the research team. I give permission for these individuals to have access to my records.	☐
4. I agree to my GP/responsible clinician being informed of my participation in the study and that they receive a report on the diagnostic assessment carried out.	☐
5. I agree to be contacted again in the future by members of the research team of this study and to be asked whether I would be willing to participate in another study.	☐
6. I agree that a video/audio recording of the testing session may be made for training purposes of members of the research team supervised by external experts, who are required to keep the contents confidential (This is optional).	
7. I agree to take part in the above study.	☐

Name of Participant	Date	Signature
Name of Person taking consent	Date	Signature

One form for participant, one form for researcher site file; the original to be kept in medical notes.

For peer review only - http://bmjopen.bmj.com/site/about/guidelines.xhtml

SPIRIT

STANDARD PROTOCOL ITEMS: RECOMMENDATIONS FOR INTERVENTIONAL TRIALS

SPIRIT 2013 Checklist: Recommended items to address in a clinical trial protocol and related documents*

Section/item	Item No	Description
**Administrative information**		
Title	1	Descriptive title identifying the study design, population, interventions, and, if applicable, trial acronym
Trial registration	2a	Trial identifier and registry name. If not yet registered, name of intended registry
	2b	All items from the World Health Organization Trial Registration Data Set
Protocol version	3	Date and version identifier
Funding	4	Sources and types of financial, material, and other support
Roles and responsibilities	5a	Names, affiliations, and roles of protocol contributors
	5b	Name and contact information for the trial sponsor
	5c	Role of study sponsor and funders, if any, in study design; collection, management, analysis, and interpretation of data; writing of the report; and the decision to submit the report for publication, including whether they will have ultimate authority over any of these activities
	5d	Composition, roles, and responsibilities of the coordinating centre, steering committee, endpoint adjudication committee, data management team, and other individuals or groups overseeing the trial, if applicable (see Item 21a for data monitoring committee)
**Introduction**		
Background and rationale	6a	Description of research question and justification for undertaking the trial, including summary of relevant studies (published and unpublished) examining benefits and harms for each intervention
	6b	Explanation for choice of comparators
Objectives	7	Specific objectives or hypotheses
Trial design	8	Description of trial design including type of trial (eg, parallel group, crossover, factorial, single group), allocation ratio, and framework (eg, superiority, equivalence, noninferiority, exploratory)

For peer review only - http://bmjopen.bmj.com/site/about/guidelines.xhtml

Methods: Participants, interventions, and outcomes

Study setting	9	Description of study settings (eg, community clinic, academic hospital) and list of countries where data will be collected. Reference to where list of study sites can be obtained
Eligibility criteria	10	Inclusion and exclusion criteria for participants. If applicable, eligibility criteria for study centres and individuals who will perform the interventions (eg, surgeons, psychotherapists)
Interventions	11a	Interventions for each group with sufficient detail to allow replication, including how and when they will be administered
	11b	Criteria for discontinuing or modifying allocated interventions for a given trial participant (eg, drug dose change in response to harms, participant request, or improving/worsening disease)
	11c	Strategies to improve adherence to intervention protocols, and any procedures for monitoring adherence (eg, drug tablet return, laboratory tests)
	11d	Relevant concomitant care and interventions that are permitted or prohibited during the trial
Outcomes	12	Primary, secondary, and other outcomes, including the specific measurement variable (eg, systolic blood pressure), analysis metric (eg, change from baseline, final value, time to event), method of aggregation (eg, median, proportion), and time point for each outcome. Explanation of the clinical relevance of chosen efficacy and harm outcomes is strongly recommended
Participant timeline	13	Time schedule of enrolment, interventions (including any run-ins and washouts), assessments, and visits for participants. A schematic diagram is highly recommended (see Figure)
Sample size	14	Estimated number of participants needed to achieve study objectives and how it was determined, including clinical and statistical assumptions supporting any sample size calculations
Recruitment	15	Strategies for achieving adequate participant enrolment to reach target sample size

Methods: Assignment of interventions (for controlled trials)

Allocation:

Sequence generation	16a	Method of generating the allocation sequence (eg, computer-generated random numbers), and list of any factors for stratification. To reduce predictability of a random sequence, details of any planned restriction (eg, blocking) should be provided in a separate document that is unavailable to those who enrol participants or assign interventions

For peer review only - http://bmjopen.bmj.com/site/about/guidelines.xhtml

Allocation concealment mechanism	16b	Mechanism of implementing the allocation sequence (eg, central telephone; sequentially numbered, opaque, sealed envelopes), describing any steps to conceal the sequence until interventions are assigned
Implementation	16c	Who will generate the allocation sequence, who will enrol participants, and who will assign participants to interventions
Blinding (masking)	17a	Who will be blinded after assignment to interventions (eg, trial participants, care providers, outcome assessors, data analysts), and how
	17b	If blinded, circumstances under which unblinding is permissible, and procedure for revealing a participant's allocated intervention during the trial
**Methods: Data collection, management, and analysis**		
Data collection methods	18a	Plans for assessment and collection of outcome, baseline, and other trial data, including any related processes to promote data quality (eg, duplicate measurements, training of assessors) and a description of study instruments (eg, questionnaires, laboratory tests) along with their reliability and validity, if known. Reference to where data collection forms can be found, if not in the protocol
	18b	Plans to promote participant retention and complete follow-up, including list of any outcome data to be collected for participants who discontinue or deviate from intervention protocols
Data management	19	Plans for data entry, coding, security, and storage, including any related processes to promote data quality (eg, double data entry; range checks for data values). Reference to where details of data management procedures can be found, if not in the protocol
Statistical methods	20a	Statistical methods for analysing primary and secondary outcomes. Reference to where other details of the statistical analysis plan can be found, if not in the protocol
	20b	Methods for any additional analyses (eg, subgroup and adjusted analyses)
	20c	Definition of analysis population relating to protocol non-adherence (eg, as randomised analysis), and any statistical methods to handle missing data (eg, multiple imputation)
**Methods: Monitoring**		
Data monitoring	21a	Composition of data monitoring committee (DMC); summary of its role and reporting structure; statement of whether it is independent from the sponsor and competing interests; and reference to where further details about its charter can be found, if not in the protocol. Alternatively, an explanation of why a DMC is not needed

	21b	Description of any interim analyses and stopping guidelines, including who will have access to these interim results and make the final decision to terminate the trial
Harms	22	Plans for collecting, assessing, reporting, and managing solicited and spontaneously reported adverse events and other unintended effects of trial interventions or trial conduct
Auditing	23	Frequency and procedures for auditing trial conduct, if any, and whether the process will be independent from investigators and the sponsor
**Ethics and dissemination**		
Research ethics approval	24	Plans for seeking research ethics committee/institutional review board (REC/IRB) approval
Protocol amendments	25	Plans for communicating important protocol modifications (eg, changes to eligibility criteria, outcomes, analyses) to relevant parties (eg, investigators, REC/IRBs, trial participants, trial registries, journals, regulators)
Consent or assent	26a	Who will obtain informed consent or assent from potential trial participants or authorised surrogates, and how (see Item 32)
	26b	Additional consent provisions for collection and use of participant data and biological specimens in ancillary studies, if applicable
Confidentiality	27	How personal information about potential and enrolled participants will be collected, shared, and maintained in order to protect confidentiality before, during, and after the trial
Declaration of interests	28	Financial and other competing interests for principal investigators for the overall trial and each study site
Access to data	29	Statement of who will have access to the final trial dataset, and disclosure of contractual agreements that limit such access for investigators
Ancillary and post-trial care	30	Provisions, if any, for ancillary and post-trial care, and for compensation to those who suffer harm from trial participation
Dissemination policy	31a	Plans for investigators and sponsor to communicate trial results to participants, healthcare professionals, the public, and other relevant groups (eg, via publication, reporting in results databases, or other data sharing arrangements), including any publication restrictions
	31b	Authorship eligibility guidelines and any intended use of professional writers
	31c	Plans, if any, for granting public access to the full protocol, participant-level dataset, and statistical code

For peer review only

For peer review only - http://bmjopen.bmj.com/site/about/guidelines.xhtml

Appendices

Informed consent materials	32	Model consent form and other related documentation given to participants and authorised surrogates
Biological specimens	33	Plans for collection, laboratory evaluation, and storage of biological specimens for genetic or molecular analysis in the current trial and for future use in ancillary studies, if applicable

*It is strongly recommended that this checklist be read in conjunction with the SPIRIT 2013 Explanation & Elaboration for important clarification on the items. Amendments to the protocol should be tracked and dated. The SPIRIT checklist is copyrighted by the SPIRIT Group under the Creative Commons "Attribution-NonCommercial-NoDerivs 3.0 Unported" license.

For peer review only

BMJ Open

Study Protocol for The Antidepressant Advisor (ADeSS): A Decision Support System for Antidepressant Treatment for Depression in UK primary care - a feasibility study

Journal:	*BMJ Open*
Manuscript ID	bmjopen-2019-035905.R2
Article Type:	Protocol
Date Submitted by the Author:	01-Apr-2020
Complete List of Authors:	Harrison, Phillippa; Centre for Affective Disorders, Department of Psychological Medicine, Institute of Psychiatry, Psychology & Neuroscience, King's College London<br>Carr, Ewan ; Department of Biostatistics and Health Informatics, Institute of Psychiatry, Psychology & Neuroscience, King's College London<br>Goldsmith, Kimberley; Department of Biostatistics and Health Informatics, Institute of Psychiatry, Psychology & Neuroscience, King's College London<br>Young, Allan; Centre for Affective Disorders, Department of Psychological Medicine, Institute of Psychiatry, Psychology & Neuroscience, King's College London<br>Ashworth, Mark; Department of Population Health Sciences, King's College London<br>Fennema, Diede; Centre for Affective Disorders, Department of Psychological Medicine, Institute of Psychiatry, Psychology & Neuroscience, King’s College London<br>Barrett, Barbara; Department of Health Services & Population Research, Institute of Psychiatry, Psychology & Neuroscience, King's College London<br>Zahn, Roland; Centre for Affective Disorders, Department of Psychological Medicine, Institute of Psychiatry, Psychology & Neuroscience, King’s College London
**Primary Subject Heading**:	Mental health
Secondary Subject Heading:	Evidence based practice
Keywords:	Depression & mood disorders < PSYCHIATRY, PRIMARY CARE, Adult psychiatry < PSYCHIATRY

SCHOLARONE™
Manuscripts

I, the Submitting Author has the right to grant and does grant on behalf of all authors of the Work (as defined in the below author licence), an exclusive licence and/or a non-exclusive licence for contributions from authors who are: i) UK Crown employees; ii) where BMJ has agreed a CC-BY licence shall apply, and/or iii) in accordance with the terms applicable for US Federal Government officers or employees acting as part of their official duties; on a worldwide, perpetual, irrevocable, royalty-free basis to BMJ Publishing Group Ltd ("BMJ") its licensees and where the relevant Journal is co-owned by BMJ to the co-owners of the Journal, to publish the Work in this journal and any other BMJ products and to exploit all rights, as set out in our licence.

The Submitting Author accepts and understands that any supply made under these terms is made by BMJ to the Submitting Author unless you are acting as an employee on behalf of your employer or a postgraduate student of an affiliated institution which is paying any applicable article publishing charge ("APC") for Open Access articles. Where the Submitting Author wishes to make the Work available on an Open Access basis (and intends to pay the relevant APC), the terms of reuse of such Open Access shall be governed by a Creative Commons licence – details of these licences and which Creative Commons licence will apply to this Work are set out in our licence referred to above.

Other than as permitted in any relevant BMJ Author's Self Archiving Policies, I confirm this Work has not been accepted for publication elsewhere, is not being considered for publication elsewhere and does not duplicate material already published. I confirm all authors consent to publication of this Work and authorise the granting of this licence.

For peer review only

Study Protocol for The Antidepressant Advisor (ADeSS): A Decision Support System for Antidepressant Treatment for Depression in UK primary care - a feasibility study

Short Title: The Antidepressant Advisor

Phillippa Harrison[1], Ewan Carr[2], Kimberley Goldsmith[2], Allan H. Young[1], Mark Ashworth[3], Diede Fennema[1], Barbara Barrett[4], Roland Zahn[1]

[1]Centre for Affective Disorders
Department of Psychological Medicine
Institute of Psychiatry, Psychology & Neuroscience
King's College London
16 De Crespigny Park
London SE5 8AF

[2]Department of Biostatistics and Health Informatics
Institute of Psychiatry, Psychology & Neuroscience
King's College London
16 De Crespigny Park
London SE5 8AF

[3]Department of Population Health Sciences,
King's College London
Guy's Campus
London SE1 1UL

[4]Department of Health Services & Population Research,
Institute of Psychiatry, Psychology & Neuroscience
King's College London
David Goldberg Centre
De Crespigny Park
London SE5 8AF

Corresponding author:
Dr Roland Zahn
Centre for Affective Disorders
Department of Psychological Medicine
Institute of Psychiatry, Psychology & Neuroscience
King's College London
PO72, De Crespigny Park, London SE5 8AF
Telephone: +44 (0)20 7848 0348
E-mail: roland.zahn@kcl.ac.uk

Word count: 4139

For peer review only

Acknowledgements

This paper represents independent research funded by the National Institute for Health Research (NIHR) research for patient benefit scheme. This paper represents independent research part funded (KG, EC) by the National Institute for Health Research (NIHR) Biomedical Research Centre at South London and Maudsley NHS Foundation Trust and King's College London. The views expressed are those of the authors and not necessarily those of the NHS, the NIHR or the Department of Health and Social Care. DF's PhD is funded by the Medical Research Council Doctoral Training Partnership (project reference: 2064430). The authors would like to acknowledge the support provided to the study by the South London Clinical Research Network and sponsorship by Lambeth CCG. Additionally, the authors wish to thank the following members of the Trial Steering Committee who have dedicated their time and provided valuable input to the study: Dr Daniel Dietch, Dr Victoria Cornelius, Evelyn London and Dr Sarah Markham. We are also grateful to EMIS PLC with whom we have designed the software implementation of our Antidepressant Advisor decision support tool and to Alloc Modulo LTD with whom we have developed the accompanying MooDoC mobile app.

For peer review only

For peer review only - http://bmjopen.bmj.com/site/about/guidelines.xhtml

Abstract

Introduction: The Antidepressant Advisor Study is a feasibility trial of a computerised decision-support tool which uses an algorithm to provide antidepressant treatment guidance for General Practitioners (GPs) in the UK primary care service. The tool is the first in the UK to implement national guidelines on antidepressant treatment guidance into a computerised decision support tool.

Methods and analysis: The study is a parallel group, cluster-randomised controlled feasibility trial where participants are blind to treatment allocation. GPs were assigned to two treatment arms: 1) treatment as usual, 2) computerised decision support tool to assist with antidepressant choices. The study will assess recruitment and lost to follow-up rates, GP satisfaction with the tool, and impact on health service use. A meaningful long-term roll-out unit cost will be calculated for the tool and service use data will be collected at baseline and follow-up to inform a full economic evaluation of a future trial.

Ethics and dissemination: The study has received NHS ethical approval from the London - Camberwell St Giles Research Ethics Committee (ref:17/LO/2074). The trial was pre-registered in the Clinical Trials.gov registry. The results of the study will be published in a pre-publication archive within one year of completion of the last follow-up assessment.

Trial registration number: ClinicalTrials.gov Identifier: NCT03628027

Strengths and limitations of this study:

- A strength of the study is integration of a decision support tool into an existing UK primary care computer system to reduce the burden on GPs of adopting a new treatment system
- A further strength is that the study allows patients to be treated by their regular GP practice which reduces the burden on patients
- A limitation of the study is the potential for selection bias towards those with less severe depression who are well enough to attend study assessments

For peer review only - http://bmjopen.bmj.com/site/about/guidelines.xhtml

Introduction

Background & Rationale

Depression is a leading cause of disability world-wide [1] with approximately two million adults in the UK experiencing an episode each week [2]. Population-based studies show that only 50% of people with major depressive disorder (MDD) are treated [3 4] and more than half of these receive inadequate treatment [4]. Furthermore, less than one third of patients fully recover after treatment with a standard antidepressant, such as citalopram [5], and only 40% of patients recover after accessing the National Health Service (NHS) Improving Access to Psychological Therapies (IAPT) programme [6]. The UK National Institute for Health and Care Excellence (NICE) recommends three first-line antidepressants with similar mechanism of action (fluoxetine, sertraline, and citalopram) [7]. Second- and third-line treatments are recommended by NICE, but, due to a lack of sequenced guidance, it is unclear whether and when GPs should prescribe them [7]. Overall, unlike for the management of other long-term conditions, there is no structured disease management for antidepressant prescribing in UK primary care [8 9] and patients' access to second- and third-line antidepressants is variable. National prescription data show frequent and rising use of certain second-line antidepressants such as venlafaxine and mirtazapine without clear decision strategies [10]. A lack of structure in treatment decision-making and poor implementation of existing guidelines could exacerbate the suffering caused by insufficiently treated depression [4]. Enhanced depression care has been called for and entails tailoring treatment to measured outcomes [9].

One way to provide structured treatment guidelines is through algorithms which incorporate various patient characteristics and allocate treatments most likely to be effective. To the authors' knowledge, there is no scientifically evaluated and pragmatic antidepressant decision support tool in UK primary care. This is based on a literature search conducted in September 2019 in the ISRCTN, Eudra CT, Clinical Trials.gov, PubMed and Web of Knowledge databases using the key words: ("depression or depressive") AND "decision" AND ("computer or algorithm") AND "antidepressant". The search returned four relevant studies which employed a computer-based algorithm to help make decisions about antidepressant treatments [11-14].

Out of three completed studies, two found evidence for the effectiveness of algorithm support compared to treatment-as-usual. Adli et al. (2017) [13] found shorter time to remission and fewer medication switches using an algorithm compared to computerised support and treatment-as-usual for depression treatment. Kurian et al. (2009) [11] assigned private GPs and their MDD

For peer review only - http://bmjopen.bmj.com/site/about/guidelines.xhtml

patients to active computerised decision support for antidepressants based on the 1999 Texas Medication Algorithm Project (TMAP) [15] and treatment-as-usual. The active group showed a 41% response rate to treatment vs. 26% in the treatment-as-usual group. However, Rollman et al. (2002) [12] reported no difference in depressive symptoms after six months between active decision-support, non-specific prompting that depression needs treatment and treatment-as-usual. The ongoing Assurex Health Inc study [14] uses a genetic testing decision support tool to predict differential treatment response to treatments for depression.

Overall, all of the above studies were conducted outside of the UK, often in private healthcare settings which are difficult to compare with the UK NHS. Several of the support tools did not provide specific treatment sequences, only broad recommendations to change medication. Furthermore, some of the studies could not translate recommendations into algorithms to be used by GPs so that researchers had to manually provide GPs with advice reports. This creates a complicated treatment process and it seems unlikely that the support tools could provide ongoing recommendations to prescribers after initial treatment recommendations. Moreover, Kurian et al. (2009) [11] and Rollman et al. (2002) [12] employed algorithms that are now outdated and lacked integration of recent evidence on superiority of specific novel antidepressants in terms of tolerability and efficacy. All of the tools lacked integration into existing electronic healthcare record systems that GPs are familiar with. The NICE guidelines provide useful MDD treatment advice for GPs but a scarcity of studies on complex interventions using sequenced treatment algorithms has meant that the guidelines are unclear about how many switches between individual antidepressants are recommended before seeking specialist advice on other strategies i.e. augmentation. The seminal STAR*D study investigating such a sequenced treatment algorithm has used augmentation strategies that according to NICE should not be carried out by GPs [16].

Therefore, the aim of the current study is to assess the feasibility of a future definitive randomised controlled trial to test the efficacy and cost-effectiveness of a decision tool incorporating an algorithm to advise GPs on antidepressant prescribing for patients who have not responded to first-line treatments. In contrast to earlier work, the treatment algorithm is based on NICE guidelines including the latest health technology appraisals. If successful, the present study will therefore enable the first investigation into whether a sequenced treatment algorithm that relies on well-tolerated single antidepressants is effective and cost-effective. The authors have gained support from one of the leading UK providers of primary care electronic

For peer review only - http://bmjopen.bmj.com/site/about/guidelines.xhtml

record systems (EMIS group) who have implemented the algorithm into their software. The tool will be designed to be easy to use and to save time for GPs who are often limited to 10-minute appointments.

Study Objectives

1) To assess the feasibility of a future confirmatory trial investigating the first computerised decision support tool for antidepressant treatment in UK primary care by:
    a. estimating lost to follow-up rates;
    b. estimating GP adherence to the algorithm and patient adherence to prescribed medications;
    c. determining the number of GP practices willing to recruit patients for the study (determined by the Clinical Research Network (CRN) who will approach all practices in the participating Clinical Comissioning Groups (CCG));
    d. estimating participant recruitment rates per GP;
    e. estimating GP satisfaction with the decision tool.
2) To provide initial standard deviation estimates and intra-class correlation coefficients for computing effect size estimates for a larger confirmatory trial.
3) To collect health economic estimates of the roll-out cost of the intervention and changes in service use associated with the tool, including psychiatric referrals to mental health teams and/or the study psychiatrist.

Methods

Trial Design

The study design is a single-blinded cluster-randomised controlled clinical trial, where the GP practice is the cluster/randomisation unit. Considering complex intervention guidance [17], the study is designed as a feasibility study of the decision support tool to provide estimates of the unknown variables needed to plan a subsequent larger trial.

Study Setting

The study is based in UK primary care, recruiting GPs and patients from South London primary care practices. Patients will be assessed at the Institute of Psychiatry, Psychology and

For peer review only - http://bmjopen.bmj.com/site/about/guidelines.xhtml

Neuroscience in South London and treated at their usual GP practice. The study start date was August 2018 and the planned end date is July 2021.

Participants

Eligibility criteria for patients

Inclusion criteria: age ≥ 18; at least moderately severe major depressive syndrome on Patient Health Questionnaire (PHQ-9; score≥15) [18]; no plans to change GP practice; able to complete self-report scales orally or in writing; no previous prescription of mirtazapine or vortioxetine; and early treatment resistance. The latter is defined as (i) current or recent prescription (in previous 2 months) of any of the following antidepressants: citalopram, fluoxetine, sertraline, escitalopram, paroxetine, venlafaxine, or duloxetine; and (ii) previous prescription of at least one other antidepressant from the same list.

Exclusion criteria: inability to consent to the study; unstable medical condition; currently being treated by mental health specialist; high suicide risk (Mini International Neuropsychiatric Interview (MINI suicidality screen)) [19]; past diagnosis of schizophrenia or schizo-affective disorder; current psychotic symptoms (three clinical screening questions to exclude schizophreniform disorders [20 21]); bipolar disorder (WHO Composite International Diagnostic Interview (CIDI) screening scale for bipolar disorder [22]); currently at risk of being violent; drug or alcohol abuse over the last six months (Primary Care Evaluation of Mental Disorders (PRIME-MD [23]), modified to screen for drug abuse); breastfeeding/ within six months of giving birth; and both escitalopram and sertraline have already been prescribed.

Participant timeline

Potential participants will be identified using an EMIS eligibility tool to search participating GP practice medical records for patients meeting inclusion criteria (see Supplementary file 1). Interested participants will complete a pre-screening assessment, followed by a screening assessment including a clinical evaluation to determine full eligibility. The results of the screening assessment will be shared with GPs and patients. Inclusion decisions will not be made by GPs to help mitigate against selection biases and will be decided by the research team after the screening assessment. After being enrolled, patients receive access to a secure mobile phone app developed in the study. Eligible patients will undergo treatment for depression over 14 weeks with their GPs. In both arms, patients will be seen by their GP who will review and

For peer review only - http://bmjopen.bmj.com/site/about/guidelines.xhtml

monitor treatment. Patients' participation will last for approximately 15-18 weeks after their screening assessment, at which point their follow-up assessment will be conducted. See Table 1 below for participant timeline.

Table 1- Participant Timeline

	Participants identified by EMIS search tool	Consent for contact	Initial Pre-screening (electronic, letter, or phone)	Pre-Trial Screening Assessment in person	Treatment Sessions arranged by GP as soon as possible after pre-trial assessment with no fixed number over 14 weeks	Post-Trial assessment in person after 15-18 weeks since Pre-Trial assessment
Participants contacted about study	X					
Reply by letter slip, phone, text or email		X				
Oral/electronic or written pre-screening informed consent			X			
Introduction to the study			X			
Assessment of eligibility			X	X		
Written informed consent				X		
Clinical assessment & neuro-psychological tests				X		X
Trial intervention delivered by GPs					X	
Mobile app/ phone weekly assessment					X	

Interventions

Participants meeting the eligibility criteria will participate in the intervention arm to which their GP practice is randomised:

For peer review only

Arm 1: Treatment-as-usual (TAU). Treatment as GPs would usually deliver with no decision support and no constraints (these GPs will not be aware of the rules of the treatment algorithm used in the other group nor will they be able to access it);

Arm 2: Decision Support Tool. Treatment using the decision support tool to assist GPs with antidepressant prescriptions, prompting GPs to review patients' medications and change them if ineffective. The algorithm and technical requirements of the tool are described below.

An EMIS decision support module was developed which provides suggestions for antidepressant prescribing after highlighting the importance of non-pharmacological strategies such as psychological therapy or exercise. The treatment recommendations are based on NICE guidelines, British Association for Psychopharmacology guidelines [24] and Maudsley prescribing guidelines [25]. The algorithm is designed to incorporate patient preferences, ensure maximum prescribing safety and offer guidance on antidepressant monotherapy (avoiding combinations). The NICE guidelines state that GPs should not normally initiate combinations without consulting a psychiatrist first [7]. See Figure 1 for stepped recommendations provided in the tool; for further detail see Supplementary file 1.

(please insert Figure 1 here)

The module is implemented only for those GP practices taking part in the study and only for participating patients at those practices. GPs will use the EMIS module during patient appointments by entering patients' Maudsley Modified Patient Health Questionnaire (MM-PHQ-9) [26] scores of their depressive symptoms over the last week from the study mobile app. The tool will offer treatment advice on whether a patient's antidepressant should be maintained or changed and what it should be changed to. The advisor tool is used for every depression review appointment over the 14-week study duration, with no minimum number of appointments.

GPs in both conditions will receive training by the study Principal Investigator (PI) on study procedures including adverse event reporting and incorporating patients' mobile app data. Training will be tailored to treatment arm so that GPs in the decision support tool arm will be trained in how to use the EMIS module to guide treatment decisions whereas GPs in the TAU arm will be advised to use their clinical judgement as usual for treatment.

For peer review only - http://bmjopen.bmj.com/site/about/guidelines.xhtml

Adherence

Adherence to the prescribed drugs in both arms will be monitored using the available tools on EMIS as well as the mobile app. Patients will be withdrawn from the antidepressant early in the event of a serious or medically important adverse event considered by the GP to be related to the intervention, hypomania, or a significant deterioration of symptoms requiring referral to secondary care. GP adherence to the algorithm will be measured after GPs have completed their participation in the study with a self-report questionnaire and information from EMIS.

Outcomes

Feasibility outcome measures:

1) Lost to follow-up rates;
2) GP adherence to the algorithm for each completed patient rated by a trial clinician (0 – 3 for none, less than 50%, 50% or more or 100% of recommended steps implemented);
3) Average patient adherence to prescribed medications based on EMIS electronic prescribing records;
4) Adverse Event (AE) and Serious Adverse Event (SAE) rates (grade and relationship to intervention);
5) Patient adherence to GP attendance measured by % of attended GP visits out of scheduled visits on EMIS over treatment period;
6) Recruitment rates;
7) Average GP satisfaction with decision support tool (intervention arm; after GP completion of study);
8) DSM-IV Social and Occupational Functioning Assessment Scale (SOFAS) scale of psychosocial functioning (on final visit, whilst modelling baseline score) [27];
9) Maudsley Visual Analogue Mood Scale (on final visit, whilst modelling baseline score);

This section describes outcomes that will be measured in this feasibility trial to provide estimates (e.g. effect size, standard deviation) needed for sample size calculations for a future confirmatory trial. These outcomes will not be used for inferential comparisons between trial arms.

Primary clinical outcomes for a future confirmatory trial:

For peer review only - http://bmjopen.bmj.com/site/about/guidelines.xhtml

10) Self-rated Quick Inventory of Depressive Symptomatology sum score (QIDS-SR16) [28] at final visit, adjusting for baseline score;

Secondary clinical outcome measures for a future confirmatory trial:

11) Depressive symptoms assessed by the Montgomery–Asberg Depression Rating Scale (MADRS) at follow-up assessment, adjusting for baseline score [29];

12) Clinical Global Impression scale; change between baseline and follow-up assessment (final visit) [30];

13) Generalised Anxiety Disorder (GAD-7) scale at follow-up assessment, adjusting for baseline score [23];

14) Body Mass Index at follow-up assessment adjusting for baseline score;

Exploratory clinical outcome measures (self-report via mobile app)

15) Average score for medication side effects on Frequency, Intensity and Burden of Side Effects Ratings (FIBSER) [31];

16) Average % of adherence to prescribed antidepressant medication;

17) Average MM-PHQ-9 scores in last 2 weeks (at follow-up, whilst modelling first 2 weeks as baseline average) [26];

Health Economic measures:

18) Service Use as determined on EMIS including psychiatric referrals and referrals to study psychiatrist, as well as time to psychiatric referral; also primary care consultation rates;

19) Service Use; self-reported using a modified version of the Adult Service Use Schedule (AD-SUS) [32];

20) Quality of life using the EQ-5D-3L [33] - the standard measure recommended by NICE for cost-effectiveness analyses.

Sample size

Each practice will be asked to enrol approximately 8-11 participants. The study aims to enrol 86 participants assuming the same lost to follow-up rate as in Kurian et al.'s (2009) study [11](18%), giving a final sample size of 70 (35 in each group as recommended [34]). This will enable estimation of the lost to follow-up rate within a 95% confidence interval of ± 8% [35]. Kurian et al. [11] did not provide effect size estimates so the present study has been designed to provide

For peer review only - http://bmjopen.bmj.com/site/about/guidelines.xhtml

means and standard deviations, as well as confidence intervals for measures of change on the primary outcome measure (QIDS-SR16 [28]) as recommended for feasibility trials [34].

Recruitment of GP Practices

The study aims to recruit 8-20 GP practices. Only one GP per practice will be recruited to the study to avoid communication about treatment allocation between GPs. In the event of a GP leaving a participating practice, an alternative GP from the same practice will replace them.

Enrolment of Participants

Patients will be enrolled after GPs are randomised. An EMIS eligibility tool developed for the study will identify potential participants from each practice meeting the inclusion/exclusion criteria (see Supplementary file 1 for further details). Practice/CRN staff will run the EMIS search and send eligible patients a study advert and request for consent for contact via post.

Assignment of Interventions

Allocation

Sequence Generation: The randomisation service will be provided by the King's Clinical Trials Unit (KCTU) in accordance with a standard operating procedure and held on a secure server. GP practices will be randomised in pairs with a 1:1 allocation to either intervention or TAU using block randomisation with random permuted blocks of block size two. This will help maintain allocation concealment while ensuring a similar number of practices are allocated to each arm.

Concealment Mechanism: KCTU will send the randomisation outcome to unblinded researchers only to ensure researchers conducting assessments remain blind. The randomisation details will be kept on a password-protected network drive only accessible by the PI.

Blinding

Patients, researchers assessing observer-rated outcomes and the senior statistician will be blind to treatment allocation. All other members of the study team, including the junior statistician, and GPs will be unblinded. Unblinding incidents will be recorded in the trial master file and reported to the Trial Steering Committee (TSC; see Data Monitoring section). Unblinded assessments will be retained in the study. If a researcher becomes unblinded prior to completing assessments, an alternative researcher will conduct future assessments with patients from that

For peer review only

For peer review only - http://bmjopen.bmj.com/site/about/guidelines.xhtml

practice in order to conduct unbiased assessments. There is no requirement for an emergency unblinding procedure, as the PI and GPs are not blinded.

Data Collection Methods

The pre-screening assessment is conducted online using a survey software (alternatively over the telephone or via postal survey) and includes the PRIME-MD (self-report) [23] to assess depressive symptoms and alcohol abuse and modified to screen for drug abuse, the CIDI (self-report) for bipolar disorder [22], questions to exclude schizophreniform disorders and psychotic depression [20 21], and pregnancy in women. Eligible participants are invited to a face-to-face screening assessment which includes depression history, treatment history, medical history, MINI suicidality screen [19], DSM-5 Structured Clinical Diagnostic Interview [27], Psychiatric Family History Screen [36], AMDP Psychopathology Interview questions of depression items [37 38], Addenbrooke's Cognitive Examination-III in patients over 50 to detect early Alzheimer's disease [39], Longitudinal Interval Follow-up Interview (LIFE) [40], and the Young Mania Rating Scale (YMRS) [41]. Participants' medical records will be checked to confirm medical details. Participants will use the mobile app for the 14-week study duration to enter weekly MM-PHQ-9 ratings [26], hypomanic symptoms [22], FIBSER medication side effects [31], medication changes and self-blame-related action tendencies such as to what extent participants would feel like hiding or creating a distance from themselves (using two questions developed in Dr Zahn's research lab). A daily question will ask about medication adherence. Alternatively, participants can opt to provide this data at weekly intervals via post or phone.

At the follow-up assessment, the study outcome measures and YMRS [41] will be repeated. The LIFE [40] will be used to determine remission and its psychiatric status rating scale used as a comparison to baseline. All assessments will be conducted by trained researchers. The research team will be trained and closely supervised, establishing sufficient inter-rater reliability on semi-structured interviews with the PI before conducting assessments independently [21].

Patient and Public Involvement

The study is supported by a service user advisory group which provides input to the study. This group meets on a regular basis and provides insight into the study design, information disseminated to patients and the burden of trial participation from the patient's perspective. At the end of the study, the service user advisory group will comment on the findings and contribute to the dissemination plan.

For peer review only - http://bmjopen.bmj.com/site/about/guidelines.xhtml

Statistical Methods

Outcomes

Categorical outcomes (e.g. loss to follow-up) will be described using frequencies and proportions. The QIDS-SR16 and other continuous outcomes will be summarised at baseline and follow-up using means and standard deviations. The GP practice intra-class correlation will be calculated for the outcome variable using one-way random effects analysis of covariance (adjusted for baseline). A preliminary analysis of the difference between the groups, as far as possible using the intention-to-treat principle, is planned. This analysis will be identified as preliminary and underpowered when published, and no p values will be provided. Continuous outcomes measured at baseline and follow-up only, such as the QIDS-SR16, will be analysed using linear regression with robust standard error calculation to account for clustering within GP practice. Continuous outcomes measured weekly will be analysed using mixed linear regression models with a random intercept for GP practice to account for clustering. Both sets of models will include treatment arm as a covariate and will adjust for baseline measure of the outcomes where appropriate. Any missing baseline data will be imputed using mean imputation [42]. Missing data in weekly outcomes will be accounted for under the missing at random assumption by using the maximum likelihood algorithm to fit the mixed models. We may consider multiple imputation for outcomes measured only at follow-up if post-randomisation variables can be quantified and are related to having missing outcomes. Adverse events (see Harms section) will be summarised separately as AEs and SAEs, by intervention group, as number of events and number of people experiencing events.

Data Monitoring

The TSC will meet bi-annually to oversee study progress and comprises the study PI, research associate, research assistants, statisticians, collaborators and service users. There are no formal criteria for terminating the trial. Informal decisions about early trial termination will be made by the TSC and sponsor should they have major concerns about safety or conduct of the trial. This would be considered in the event that extensive recurrent serious or medically important adverse events are observed, particularly if these are Suspected Unexpected Serious Adverse Reactions (SUSARs).

Harms

For peer review only - http://bmjopen.bmj.com/site/about/guidelines.xhtml

GPs participating in the study, especially in the intervention arm, are likely to change medications more often and prescribe medications which are less frequently used in primary care. All medications recommended by the decision support tool are in accordance with NICE guidance and were carefully selected for the study based on likelihood of positive effects and side effects (see Supplementary file 1). Kurian et al. (2009) [11] found their support tool to be beneficial for depression treatment, although some patients felt worse as a result of changing medications – for example, having more suicidal thoughts. Such side effects will be monitored by GPs and discussed with patients.

AEs/SAEs are defined in Supplementary file 1. AEs/SAEs will be recorded from the time the subject provides informed consent at the baseline assessment until their follow-up. GPs will record all AEs/SAEs on EMIS and they will be regularly collected from GPs by the research team and assessed. AEs/SAEs reported to the PI will be recorded on AE forms as part of the case report form using an ID number to identify the patient. AEs/SAEs will be reported bi-annually to the TSC and an unblinded sub-committee, assuming the functions of the data monitoring committee, will review the information separated by arm.

Ethics and Dissemination

Research Ethics Approval

The study has received NHS ethical approval from the London - Camberwell St Giles Research Ethics Committee (ref:17/LO/2074).

Protocol Amendments

Modifications to the protocol will be conveyed to the TSC at the planning stage for members to provide input, then again to inform the committee of the ethics committee decision.

Consent

Participants will provide informed consent to take part in the study and are free to withdraw at any time with no reason given (see Supplementary files 1 & 2 for further detail).

Dissemination

The trial has been pre-registered on ClinicalTrials.gov (Identifier: NCT03628027). See Supplementary file 1 for further detail on dissemination.

For peer review only - http://bmjopen.bmj.com/site/about/guidelines.xhtml

Health economics

The study will not include a formal economic evaluation, instead the focus is on developing the tools needed to support a future trial. First, a meaningful unit cost will be calculated for our tool in collaboration with EMIS. The unit cost will consider the long-term roll-out cost of the intervention and investigate how to allocate this to an individual patient in order to carry out a future cost-effectiveness analysis. Second, the methods for the collection of service use data will be optimised, which will include a comparison of data collected from EMIS and data collected from patients via self-report.

Limitations

One limitation of the algorithm is that it does not take pharmacogenomic evidence into account which could be an important refinement in the future, should pharmacogenomic testing become implemented into NHS routine care. For example, both CYP2D6 and CYP2C19 gene polymorphisms affect the liver metabolism of sertraline, escitalopram and citalopram, as well as paroxetine and fluvoxamine [43]. Therefore one could argue if someone has not responded to or not tolerated citalopram or paroxetine, this carries a higher risk of non-tolerance or non-response of escitalopram and sertraline. Pharmacogenomic effects of liver enzymes, however, are only one of many factors which determine response and tolerance [44]. From a pragmatic perspective it is therefore worth trying escitalopram or sertraline as the first step in the algorithm which is supported by network-meta-analytical evidence[45]. CYP2D6 also plays a role in metabolising vortioxetine and mirtazapine, but pharmacogenomic guidelines have not been developed for these to our knowledge.

Contributorship Statement

PH wrote the manuscript and EC, KG, AHY, MA, DF, BB and RZ all commented significantly on drafts of the manuscript. EC and KG provided the statistical analysis plan, BB provided the health economic analysis plan and AHY, MA and RZ provided oversight on the study procedures and delivery.

Competing Interests

For peer review only

AHY is employed by King's College London as an honorary consultant in the South London and Maudsley Trust (NHS UK) and is a consultant to Johnson & Johnson and Livanova. AHY has given paid lectures and sat on advisory boards for the following companies with drugs used in affective and related disorders: Astrazenaca, Eli Lilly, Lundbeck, Sunovion, Servier, Livanova, Janssen, Allegan, Bionomics, Sumitomo Dainippon Pharma. AHY has received honoraria for attending advisory boards and presenting talks at meetings organised by LivaNova. AHY is the Principal Investigator of the following studies: Restore-Life VNS registry study funded by LivaNova, ESKETINTRD3004: "An Open-label, Long-term, Safety and Efficacy Study of Intranasal Esketamine in Treatment-resistant Depression," "The Effects of Psilocybin on Cognitive Function in Healthy Participants," and "The Safety and Efficacy of Psilocybin in Participants with Treatment-Resistant Depression (P-TRD)." AHY has received grant funding (past and present) from the following: NIMH (USA); CIHR (Canada); NARSAD (USA); Stanley Medical Research Institute (USA); MRC (UK); Wellcome Trust (UK); Royal College of Physicians (Edin); BMA (UK); UBC-VGH Foundation (Canada); WEDC (Canada); CCS Depression Research Fund (Canada); MSFHR (Canada); NIHR (UK); Janssen (UK). AHY has no shareholdings in pharmaceutical companies.

RZ is a private psychiatrist service provider at The London Depression Institute and co-investigator on a Livanova-funded observational study of Vagus Nerve Stimulation for Depression. RZ has received honorarium for talks at three medical symposia sponsored by Lundbeck as well as a talk funded by Janssen whom he collaborates with on an MRC-funded grant. KG reports grants from NIHR, Stroke association, National Institutes of Health (US) and Juvenile Diabetes Research Foundation (US) during the conduct of the study. EC reports personal fees from NIHR during the conduct of the study. BB reports grants from NIHR Research for Patient Benefit during the conduct of the study. MA, PH and DF report no competing interests.

For peer review only - http://bmjopen.bmj.com/site/about/guidelines.xhtml

Funding

The research has received funding from the National Institute for Health Research (NIHR) research for patient benefit (RfPB) (grant reference: PB-PG-0416-20039), the National Institute for Health Research (NIHR) Biomedical Research Centre at South London and Maudsley NHS Foundation Trust and King's College London and the Medical Research Council Doctoral Training Partnership (grant reference: 2064430).

Data Sharing Statement

The trial is ongoing so data sharing is not currently available.

For peer review only

References

1. WHO. The global burden of disease: 2004 Update. 2008
2. UK National Centre for Social Research. Adult Psychiatric Morbidity Survey, 2007.
3. Dormon F. Is mental health care improving?: The Health Foundation 2015.
4. Kessler RC, Berglund P, Demler O, et al. The epidemiology of major depressive disorder: results from the National Comorbidity Survey Replication (NCS-R). *JAMA* 2003;289(23):3095-105. doi: 10.1001/jama.289.23.3095
5. Trivedi MH, Rush AJ, Wisniewski SR, et al. Evaluation of outcomes with citalopram for depression using measurement-based care in STAR*D: implications for clinical practice. *Am J Psychiatry* 2006;163(1):28-40. doi: 10.1176/appi.ajp.163.1.28
6. Gyani A, Shafran R, Layard R, et al. Enhancing recovery rates: lessons from year one of IAPT. *Behav Res Ther* 2013;51(9):597-606. doi: 10.1016/j.brat.2013.06.004
7. Depression: The treatment and management of depression in adults; 2010. The British Psychological Society and The Royal College of Psychiatrists.
8. Symons L, Tylee A, Mann A, et al. Improving access to depression care: descriptive report of a multidisciplinary primary care pilot service. *The British journal of general practice : the journal of the Royal College of General Practitioners* 2004;54(506):679-83. [published Online First: 2004/09/09]
9. Von Korff M, Goldberg D. Improving outcomes in depression. *BMJ (Clinical research ed)* 2001;323(7319):948-9. [published Online First: 2001/10/27]
10. Spence R, Roberts A, Ariti C, et al. Focus On: Antidepressant prescribing: The Health Foundation & Nuffield Trust, 2014.
11. Kurian BT, Trivedi MH, Grannemann BD, et al. A computerized decision support system for depression in primary care. *Primary care companion to the Journal of clinical psychiatry* 2009;11(4):140-6. doi: 10.4088/PCC.08m00687
12. Rollman BL, Hanusa BH, Lowe HJ, et al. A randomized trial using computerized decision support to improve treatment of major depression in primary care. *Journal of general internal medicine* 2002;17(7):493-503. [published Online First: 2002/07/23]
13. Adli M, Wiethoff K, Baghai TC, et al. How effective is algorithm-guided treatment for depressed inpatients? Results from the randomized controlled multicenter German Algorithm Project 3 trial. *International Journal of Neuropsychopharmacology* 2017;20(9):721-30.
14. Inc AH. Pharmacogenomic Decision Support With GeneSight Psychotropic to Guide the Treatment of Major Depressive Disorder. *ClinicalTrialsgov* registered 2015
15. Crismon ML, Trivedi M, Pigott TA, et al. The Texas Medication Algorithm Project: report of the Texas Consensus Conference Panel on Medication Treatment of Major Depressive Disorder. *J Clin Psychiatry* 1999;60(3):142-56.
16. Rush AJ, Fava M, Wisniewski SR, et al. Sequenced treatment alternatives to relieve depression (STAR* D): rationale and design. *Controlled clinical trials* 2004;25(1):119-42.
17. MRC. Developing and evaluating complex interventions: new guidance, 2006.
18. Spitzer RL, Kroenke K, Williams JW, et al. Validation and utility of a self-report version of prime-md: The phq primary care study. *JAMA* 1999;282(18):1737-44. doi: 10.1001/jama.282.18.1737
19. Sheehan DV, Lecrubier Y, Sheehan KH, et al. The Mini-International Neuropsychiatric Interview (M.I.N.I.): the development and validation of a structured diagnostic psychiatric interview for DSM-IV and ICD-10. *J Clin Psychiatry* 1998;59 Suppl 20:22-33;quiz 34-57.
20. Green MF, Hellemann G, Horan WP, et al. From perception to functional outcome in schizophrenia: modeling the role of ability and motivation. *Archives of General Psychiatry* 2012;69(12):1216-24.
21. Lythe KE, Moll J, Gethin JA, et al. Self-blame–selective hyperconnectivity between anterior temporal and subgenual cortices and prediction of recurrent depressive episodes. *JAMA psychiatry* 2015;72(11):1119-26.

For peer review only

22. Kessler RC, Akiskal HS, Angst J, et al. Validity of the Assessment of Bipolar Spectrum Disorders in the WHO CIDI 3.0. *Journal of affective disorders* 2006;96(3):259-69. doi: 10.1016/j.jad.2006.08.018
23. Spitzer RL, Williams JW, Kroenke K, et al. Utility of a new procedure for diagnosing mental disorders in primary care: The prime-md 1000 study. *JAMA* 1994;272(22):1749-56. doi: 10.1001/jama.1994.03520220043029
24. Cleare A, Pariante CM, Young AH, et al. Evidence-based guidelines for treating depressive disorders with antidepressants: A revision of the 2008 British Association for Psychopharmacology guidelines. *Journal of psychopharmacology* 2015;29(5):459-525. doi: 10.1177/0269881115581093
25. Taylor D, Paton C, Kapur S, et al. The Maudsley prescribing guidelines in psychiatry. 12th edition. ed: Wiley 2015.
26. Walton S. Developing a novel outcome measure for depression: The Maudsley Modified Patient Health Questionnaire-9. . *Unpublished Masters dissertation, King's College London* 2019.
27. First MB SR, Gibbon M, Williams JBW. Structured Clinical Interview for DSM-IV-TR Axis I Disorders, Research Version, Patient Edition. (SCID-I/P). . *New York, NY: Biometrics Research, New York State Psychiatric Institute* 2002.
28. Rush AJ, Trivedi MH, Ibrahim HM, et al. The 16-Item Quick Inventory of Depressive Symptomatology (QIDS), clinician rating (QIDS-C), and self-report (QIDS-SR): a psychometric evaluation in patients with chronic major depression. *Biological psychiatry* 2003;54(5):573-83.
29. Montgomery SA, Åsberg M. A new depression scale designed to be sensitive to change. *British Journal of Psychiatry* 1979;134(4):382-89.
30. Busner J, Targum SD, Miller DS. The Clinical Global Impressions scale: errors in understanding and use. *Comprehensive psychiatry* 2009;50(3):257-62. doi: 10.1016/j.comppsych.2008.08.005
31. Wisniewski SR, Rush AJ, Balasubramani GK, et al. Self-rated global measure of the frequency, intensity, and burden of side effects. *Journal of psychiatric practice* 2006;12(2):71-9. [published Online First: 2006/05/27]
32. Kuyken W HR, Barrett B, BynG R, Dalgleish T, Kessler D et al. Effectiveness and cost-effectiveness of mindfulness-based cognitive therapy compared with maintenance antidepressant treatment in the prevention of depressive relapse or recurrence (PREVENT): a randomised controlled trial. . *The Lancet* 2015;386:63-73.
33. R. B. EuroQol: the current state of play. *Health Policy* 1996.;37:53-72.
34. Teare MD, Dimairo M, Shephard N, et al. Sample size requirements to estimate key design parameters from external pilot randomised controlled trials: a simulation study. *Trials* 2014;15:264. doi: 10.1186/1745-6215-15-264
35. Lwanga SK, Lemeshow S, Organization WH. Sample size determination in health studies: a practical manual. 1991
36. Weissman MM WP, Adams P, Wolk S, Verdeli H, Olfson M. . Brief screening for family psychiatric history: the family history screen. . *Arch Gen Psychiatry* 2000;57:675.
37. Fähndrich E RE. The AMDP system for the documentation of psychiatric symptoms: course and effectivity of a training seminar. *Pharmacopsychiatry* 1985;18:278-81.
38. Fähndrich E SR. Leitfaden zur Erfassung des psychopathologischen Befundes: halbstrukturiertes Interview anhand des AMDP-Systems. *Hogrefe Verlag* 1989
39. Noone P. Addenbrooke's cognitive examination-III. *Occupational Medicine* 2015;65(5):418-20.
40. Keller MB, Lavori PW, Friedman B, et al. The Longitudinal Interval Follow-up Evaluation. A comprehensive method for assessing outcome in prospective longitudinal studies. *Arch Gen Psychiatry* 1987;44(6):540-8.
41. Young RC, Biggs JT, Ziegler VE, et al. A rating scale for mania: reliability, validity and sensitivity. *Br J Psychiatry* 1978;133:429-35.
42. White IR, Thompson SG. Adjusting for partially missing baseline measurements in randomized trials. *Statistics in medicine* 2005;24(7):993-1007. doi: 10.1002/sim.1981

For peer review only

43. Hicks JK, Bishop JR, Sangkuhl K, et al. Clinical Pharmacogenetics Implementation Consortium (CPIC) guideline for CYP2D6 and CYP2C19 genotypes and dosing of selective serotonin reuptake inhibitors. *Clinical Pharmacology & Therapeutics* 2015;98(2):127-34.
44. Bousman CA, Forbes M, Jayaram M, et al. Antidepressant prescribing in the precision medicine era: a prescriber's primer on pharmacogenetic tools. *BMC psychiatry* 2017;17(1):60.
45. Cipriani A, Furukawa TA, Salanti G, et al. Comparative efficacy and acceptability of 12 new-generation antidepressants: a multiple-treatments meta-analysis. *Lancet* 2009;373(9665):746-58. doi: 10.1016/S0140-6736(09)60046-5

For peer review only

Figure 1-Decision chart for decision support tool

For peer review only

For peer review only - http://bmjopen.bmj.com/site/about/guidelines.xhtml

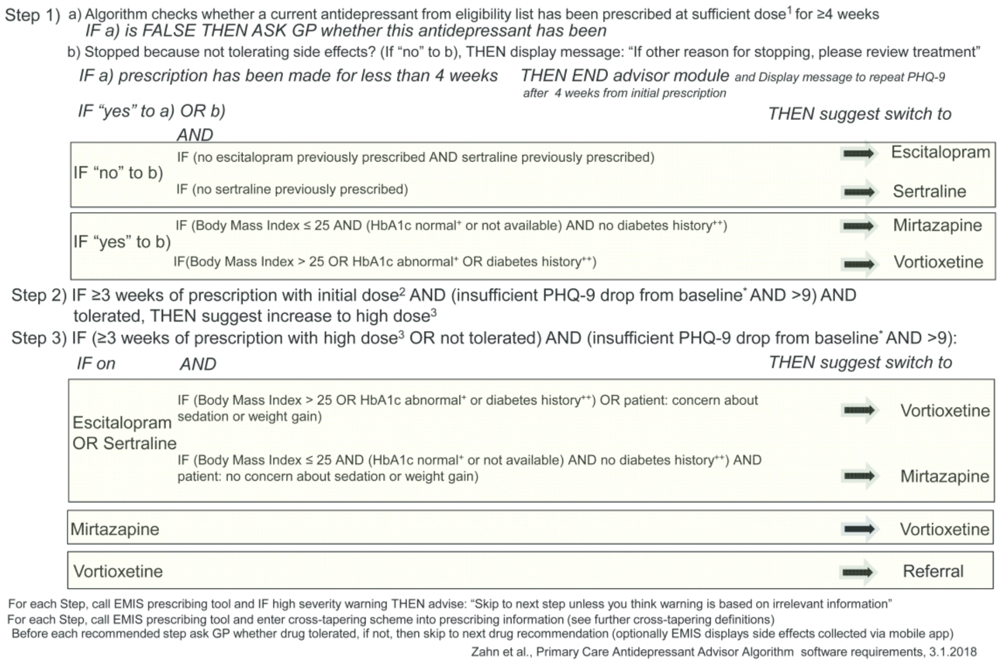

Figure 1-Decision chart for decision support tool

197x130mm (600 x 600 DPI)

SUPPLEMENTARY FILE 1

Study Protocol for The Antidepressant Advisor (ADeSS): A Decision Support System for Antidepressant Treatment for Depression in UK primary care - a feasibility study

Short Title: The Antidepressant Advisor

Supplementary Methods

EMIS Eligibility Tool Criteria

1) current or recent prescription (in the last 2 months) of any of the following antidepressants listed: citalopram, fluoxetine, sertraline, escitalopram, paroxetine, venlafaxine, or duloxetine
2) previous prescription of at least one other antidepressant out of the same list of antidepressants
3) exclude patients for whom both escitalopram and sertraline have already been prescribed
4) no previous prescription of mirtazapine or vortioxetine
5) no mania/hypomania/bipolar (or synonyms), psychosis/schizophrenia (or synonyms), or drug abuse/misuse/dependence (or synonyms), or alcohol abuse/misuse/dependence or synonyms
6) age≥18 years
7) not pregnant
8) not given birth in last 6 months.

Decision Support Tool Rationale & Details

The decision chart for the decision support tool is presented in Figure 1 below. A network meta-analysis, not including vortioxetine, showed that sertraline and escitalopram are superior in terms of acceptability and efficacy compared to other antidepressants [1]. Since sertraline is among the first–line options recommended by NICE, if patients tolerate serotonin reuptake inhibitors (SSRI), the algorithm starts with recommending escitalopram for those patients where two NICE recommended first-line treatments have failed including sertraline, but starts with sertraline should it not have been prescribed before. For patients who have not tolerated SSRIs (verified by GP), the algorithm makes alternative suggestions. Mirtazapine is among the most efficacious antidepressants [1] and has a different side effect profile to SSRIs which is why it is suggested for these patients. Because of its 75% risk in inducing weight gain and thereby increasing the risk of metabolic syndrome[2], the algorithm suggests

For peer review only - http://bmjopen.bmj.com/site/about/guidelines.xhtml

vortioxetine in those patients who are at an increased risk of metabolic syndrome (Body Mass Index(BMI)>25, HbA1c increased), because of its low weight-gain-induction risk[3]. Vortioxetine has SSRI properties, but is less likely to induce typical side effects, apart from nausea [3]. A recent NICE health technology appraisal [4]has given a positive recommendation for using vortioxetine in patients whose depressive episode has responded inadequately to two antidepressants. The REVIVE trial [3] showed the benefits of vortioxetine in patients with current MDD who had not responded to SSRI or dual-reuptake inhibitor monotherapy. The appraisal also concluded that vortioxetine may have a better overall safety profile than other antidepressants. For those patients tolerating SSRIs who do not respond to escitalopram and are at an increased metabolic syndrome risk, the algorithm therefore suggests vortioxetine.

Venlafaxine, which is among the most efficacious medications[1], was omitted from the algorithm, because of its higher risk of death in overdose which limits its use in primary care according to the Medicines and Healthcare Regulatory Agency [5]guidance and most GPs we discussed this with are not prepared to prescribe it at the higher doses at which it acts as a noradrenaline reuptake inhibitor. Duloxetine was excluded as an alternative as it was less efficacious in a network meta-analysis [1].

For escitalopram non-responders who are at low metabolic syndrome risk, the algorithm suggests mirtazapine as a next step. For mirtazapine non-responders, a switch to vortioxetine is suggested as a next step. For vortioxetine non-responders during the second or third step, a psychiatric referral to the study psychiatrist is suggested, who could then recommend higher side effect burdened alternatives such as high-dose venlafaxine or augmentation strategies according to NICE guidelines. Agomelatine has been omitted from the algorithm despite its suitability for patients who are not tolerating SSRIs, because there is no positive HTA recommendation for it and because it requires liver enzyme checks [6].

Data Management

Data will be entered electronically into a password-protected database and stored separately from identifiable information. Data validation will be applied to ensure that entered data follows the correct format and is within a valid range. Data will be stored electronically in the master folder and will only be accessed by members of the research team. Data will be

For peer review only - http://bmjopen.bmj.com/site/about/guidelines.xhtml

backed up by the research associate every time the database is added to on an external hard drive.

Harms

An adverse event (AE) is any untoward medical occurrence in a patient or healthy volunteer, which does not necessarily have a causal relationship with the study procedures. A Serious Adverse Event (SAE) is any undesirable medical occurrence in a participant that is life threatening, requires hospitalisation, results in persistent/significant disability/incapacity, causes a congenital anomaly/ birth defect or results in death.

These events may be spontaneously revealed by the subject or observed by the GP and will be recorded regardless of whether the GP believes them to be related to the intervention. The following aspects of AEs/SAEs will be assessed: symptom, possible diagnosis or syndrome, date and duration of AE/SAE, causal relationship with investigation (definite / probable / possible / unlikely / not related), outcome (resolved / resolving / not resolved / resolved with sequelae / fatal / unknown), treatment of event, if applicable (e.g. any medication as well as any other medical measures), actions taken regarding assessment.

GPs will be asked to report SAEs within 24 hours to Lambeth CCG and the PI via email or phone without details which are then followed up with a more detailed report. SAEs which according to the GP are clearly unrelated to the study intervention (e.g. hospitalization for an unrelated illness) and AEs expected when treating depression[i], even if there is a possible relation to the intervention, are exempt from this immediate reporting and will instead be reported together with other AEs at the earliest opportunity (at least quarterly). An executive committee formed of two members of the TSC (one independent) will discuss any AEs/SAEs prior to each TSC then report any concerns back to the TSC. Blinded members of the TSC will be asked to leave the room for the discussion of AEs/SAEs in order to remain blinded. Medically important events or SAEs will be assessed by a medically qualified doctor in the study team and discussed with the TSC. If possible, blinded doctors will assess serious or medically important events, whereas other AEs may be classified by a non-blinded doctor. Classification into SUSAR vs. expected ones will be based on the most updated versions of the summary product characteristics of the prescribed drugs on https://www.medicines.org.uk/emc/. SUSARs would be reported to the sponsor and ethics

For peer review only - http://bmjopen.bmj.com/site/about/guidelines.xhtml

committee. GPs will be asked to document all other AEs and reasons for withdrawing from the study on EMIS.

Consent

Consent and screening will be carried out in two stages. First, patients identified by the EMIS eligibility tool will be asked to provide written consent to participate in the online pre-screening assessment, with phone and postal options also offered. Second, patients found to be eligible at pre-screening will be invited to the baseline assessment where they will be asked to provide written consent to participate in the feasibility trial. Each participant will receive an oral and written explanation of the purposes, procedures, and risks of this study. Written consent will be obtained after explaining the goals, procedures, risks (and discomforts), benefits, and nature of the study to patients. Only adult volunteers and patients who are capable of understanding the study procedures will be allowed to sign the consent forms.

Participants can withdraw from the trial at any time and this will be made clear in the information sheets and consent forms. The reason for withdrawal does not need to be provided.

Dissemination Policy

Reporting bias will be avoided by a priori registration in a public trial register in addition to publication of the current trial protocol as the research is ongoing. All members of the TSC will have the opportunity to review and have input into the current protocol. The results of the study will be published in a pre-publication archive within one year after completion.

For peer review only

For peer review only - http://bmjopen.bmj.com/site/about/guidelines.xhtml

References

1. Cipriani A, Furukawa TA, Salanti G et al. Comparative efficacy and acceptability of 12 new-generation antidepressants: a multiple-treatments meta-analysis. Lancet 2009;373(9665):746-58.
2. Masand PS, Gupta S. Long-term side effects of newer-generation antidepressants: SSRIS, venlafaxine, nefazodone, bupropion, and mirtazapine. Ann Clin Psychiatry 2002;14(3):175-82.
3. Montgomery SA, Nielsen RZ, Poulsen LH, Häggström L. A randomised, double-blind study in adults with major depressive disorder with an inadequate response to a single course of selective serotonin reuptake inhibitor or serotonin–noradrenaline reuptake inhibitor treatment switched to vortioxetine or agomelatine. Human Psychopharmacology: Clinical and Experimental 2014;29(5):470-482.
4. NICE. Vortioxetine for treating major depressive episodes - Technology appraisal guidance. 2015.
5. (MHRA). MaHRA. Selective serotonin reuptake inhibitors (SSRIs) and serotonin and noradrenaline reuptake inhibitors (SNRIs): use and safety. . 2014.
6. Agomelatine (Valdoxan): risk of liver toxicity. . Medicines and Healthcare Regulatory Agency (MHRA). 2014.

[i] These include non-fatal suicide attempt, non-suicidal self-harm-related injury, non-fatal traffic accident under medication influence, treatment-emergent [hypo-]mania with associated non-fatal complications, non-fatal somatic or anxiety-related complaints leading to A&E visits).

For peer review only

SUPPLEMENTARY FILE 2

Participant Identification Number for this trial: **ADeSS-S1-**

Date of Birth:

CONSENT FORM

Study Title: Antidepressant Advisor – Study 1
Name of Chief Investigator: Dr Roland Zahn

Please initial:

1. I confirm that I have read and understand the information sheet dated 24 Jan 2018 (version 2) for the above study. I have had the opportunity to consider the information, ask questions and have had these answered satisfactorily.	☐
2. I understand that my participation is voluntary and that I am free to withdraw at any time without giving any reason, without my medical care or legal rights being affected.	☐
3. I understand that relevant sections of my medical notes and data collected during the study may be looked at by individuals from the research team. I give permission for these individuals to have access to my records.	☐
4. I agree to my GP/responsible clinician being informed of my participation in the study and that they receive a report on the diagnostic assessment carried out.	☐
5. I agree to be contacted again in the future by members of the research team of this study and to be asked whether I would be willing to participate in another study.	☐
6. I agree that a video/audio recording of the testing session may be made for training purposes of members of the research team supervised by external experts, who are required to keep the contents confidential (This is optional).	
7. I agree to take part in the above study.	☐

Name of Participant	Date	Signature
Name of Person taking consent	Date	Signature

One form for participant, one form for researcher site file; the original to be kept in medical notes.

For peer review only - http://bmjopen.bmj.com/site/about/guidelines.xhtml

STANDARD PROTOCOL ITEMS: RECOMMENDATIONS FOR INTERVENTIONAL TRIALS

SPIRIT 2013 Checklist: Recommended items to address in a clinical trial protocol and related documents*

Section/item	Item No	Description
**Administrative information**		
Title	1	Descriptive title identifying the study design, population, interventions, and, if applicable, trial acronym
Trial registration	2a	Trial identifier and registry name. If not yet registered, name of intended registry
	2b	All items from the World Health Organization Trial Registration Data Set
Protocol version	3	Date and version identifier
Funding	4	Sources and types of financial, material, and other support
Roles and responsibilities	5a	Names, affiliations, and roles of protocol contributors
	5b	Name and contact information for the trial sponsor
	5c	Role of study sponsor and funders, if any, in study design; collection, management, analysis, and interpretation of data; writing of the report; and the decision to submit the report for publication, including whether they will have ultimate authority over any of these activities
	5d	Composition, roles, and responsibilities of the coordinating centre, steering committee, endpoint adjudication committee, data management team, and other individuals or groups overseeing the trial, if applicable (see Item 21a for data monitoring committee)
**Introduction**		
Background and rationale	6a	Description of research question and justification for undertaking the trial, including summary of relevant studies (published and unpublished) examining benefits and harms for each intervention
	6b	Explanation for choice of comparators
Objectives	7	Specific objectives or hypotheses
Trial design	8	Description of trial design including type of trial (eg, parallel group, crossover, factorial, single group), allocation ratio, and framework (eg, superiority, equivalence, noninferiority, exploratory)

For peer review only

Methods: Participants, interventions, and outcomes

Study setting	9	Description of study settings (eg, community clinic, academic hospital) and list of countries where data will be collected. Reference to where list of study sites can be obtained
Eligibility criteria	10	Inclusion and exclusion criteria for participants. If applicable, eligibility criteria for study centres and individuals who will perform the interventions (eg, surgeons, psychotherapists)
Interventions	11a	Interventions for each group with sufficient detail to allow replication, including how and when they will be administered
	11b	Criteria for discontinuing or modifying allocated interventions for a given trial participant (eg, drug dose change in response to harms, participant request, or improving/worsening disease)
	11c	Strategies to improve adherence to intervention protocols, and any procedures for monitoring adherence (eg, drug tablet return, laboratory tests)
	11d	Relevant concomitant care and interventions that are permitted or prohibited during the trial
Outcomes	12	Primary, secondary, and other outcomes, including the specific measurement variable (eg, systolic blood pressure), analysis metric (eg, change from baseline, final value, time to event), method of aggregation (eg, median, proportion), and time point for each outcome. Explanation of the clinical relevance of chosen efficacy and harm outcomes is strongly recommended
Participant timeline	13	Time schedule of enrolment, interventions (including any run-ins and washouts), assessments, and visits for participants. A schematic diagram is highly recommended (see Figure)
Sample size	14	Estimated number of participants needed to achieve study objectives and how it was determined, including clinical and statistical assumptions supporting any sample size calculations
Recruitment	15	Strategies for achieving adequate participant enrolment to reach target sample size

Methods: Assignment of interventions (for controlled trials)

Allocation:

Sequence generation	16a	Method of generating the allocation sequence (eg, computer-generated random numbers), and list of any factors for stratification. To reduce predictability of a random sequence, details of any planned restriction (eg, blocking) should be provided in a separate document that is unavailable to those who enrol participants or assign interventions

Allocation concealment mechanism	16b	Mechanism of implementing the allocation sequence (eg, central telephone; sequentially numbered, opaque, sealed envelopes), describing any steps to conceal the sequence until interventions are assigned
Implementation	16c	Who will generate the allocation sequence, who will enrol participants, and who will assign participants to interventions
Blinding (masking)	17a	Who will be blinded after assignment to interventions (eg, trial participants, care providers, outcome assessors, data analysts), and how
	17b	If blinded, circumstances under which unblinding is permissible, and procedure for revealing a participant's allocated intervention during the trial
**Methods: Data collection, management, and analysis**		
Data collection methods	18a	Plans for assessment and collection of outcome, baseline, and other trial data, including any related processes to promote data quality (eg, duplicate measurements, training of assessors) and a description of study instruments (eg, questionnaires, laboratory tests) along with their reliability and validity, if known. Reference to where data collection forms can be found, if not in the protocol
	18b	Plans to promote participant retention and complete follow-up, including list of any outcome data to be collected for participants who discontinue or deviate from intervention protocols
Data management	19	Plans for data entry, coding, security, and storage, including any related processes to promote data quality (eg, double data entry; range checks for data values). Reference to where details of data management procedures can be found, if not in the protocol
Statistical methods	20a	Statistical methods for analysing primary and secondary outcomes. Reference to where other details of the statistical analysis plan can be found, if not in the protocol
	20b	Methods for any additional analyses (eg, subgroup and adjusted analyses)
	20c	Definition of analysis population relating to protocol non-adherence (eg, as randomised analysis), and any statistical methods to handle missing data (eg, multiple imputation)
**Methods: Monitoring**		
Data monitoring	21a	Composition of data monitoring committee (DMC); summary of its role and reporting structure; statement of whether it is independent from the sponsor and competing interests; and reference to where further details about its charter can be found, if not in the protocol. Alternatively, an explanation of why a DMC is not needed

For peer review only - http://bmjopen.bmj.com/site/about/guidelines.xhtml

	21b	Description of any interim analyses and stopping guidelines, including who will have access to these interim results and make the final decision to terminate the trial
Harms	22	Plans for collecting, assessing, reporting, and managing solicited and spontaneously reported adverse events and other unintended effects of trial interventions or trial conduct
Auditing	23	Frequency and procedures for auditing trial conduct, if any, and whether the process will be independent from investigators and the sponsor
**Ethics and dissemination**		
Research ethics approval	24	Plans for seeking research ethics committee/institutional review board (REC/IRB) approval
Protocol amendments	25	Plans for communicating important protocol modifications (eg, changes to eligibility criteria, outcomes, analyses) to relevant parties (eg, investigators, REC/IRBs, trial participants, trial registries, journals, regulators)
Consent or assent	26a	Who will obtain informed consent or assent from potential trial participants or authorised surrogates, and how (see Item 32)
	26b	Additional consent provisions for collection and use of participant data and biological specimens in ancillary studies, if applicable
Confidentiality	27	How personal information about potential and enrolled participants will be collected, shared, and maintained in order to protect confidentiality before, during, and after the trial
Declaration of interests	28	Financial and other competing interests for principal investigators for the overall trial and each study site
Access to data	29	Statement of who will have access to the final trial dataset, and disclosure of contractual agreements that limit such access for investigators
Ancillary and post-trial care	30	Provisions, if any, for ancillary and post-trial care, and for compensation to those who suffer harm from trial participation
Dissemination policy	31a	Plans for investigators and sponsor to communicate trial results to participants, healthcare professionals, the public, and other relevant groups (eg, via publication, reporting in results databases, or other data sharing arrangements), including any publication restrictions
	31b	Authorship eligibility guidelines and any intended use of professional writers
	31c	Plans, if any, for granting public access to the full protocol, participant-level dataset, and statistical code

For peer review only

Appendices

Informed consent materials	32	Model consent form and other related documentation given to participants and authorised surrogates
Biological specimens	33	Plans for collection, laboratory evaluation, and storage of biological specimens for genetic or molecular analysis in the current trial and for future use in ancillary studies, if applicable

*It is strongly recommended that this checklist be read in conjunction with the SPIRIT 2013 Explanation & Elaboration for important clarification on the items. Amendments to the protocol should be tracked and dated. The SPIRIT checklist is copyrighted by the SPIRIT Group under the Creative Commons "Attribution-NonCommercial-NoDerivs 3.0 Unported" license.

For peer review only

For peer review only - http://bmjopen.bmj.com/site/about/guidelines.xhtml
